# A comprehensive analysis of RAS-effector interactions reveals interaction hotspots and new binding partners

**DOI:** 10.1016/j.jbc.2021.100626

**Published:** 2021-04-28

**Authors:** Soheila Rezaei Adariani, Neda S. Kazemein Jasemi, Farhad Bazgir, Christoph Wittich, Ehsan Amin, Claus A.M. Seidel, Radovan Dvorsky, Mohammad R. Ahmadian

**Affiliations:** 1 Medical Faculty, Institute of Biochemistry and Molecular Biology II, Heinrich Heine University, Düsseldorf, Germany; 2 Medical Faculty, Institute of Neural and Sensory Physiology, Heinrich Heine University, Düsseldorf, Germany; 3 Chair of Molecular Physical Chemistry, Heinrich Heine University, Düsseldorf, Germany

**Keywords:** effectors, GTPase, NORE-1, protein interactions, RAS, RASSF, RASSF1, RASSF5, RAS association domain, RAS-binding domain, AF6, ALL1-fused gene from chromosome 6, CR domain, cysteine-rich domain, ERK, extracellular signal-regulated kinase, GAP, GTPase-activating protein, GEF, guanine nucleotide exchange factor, GTP, guanosine triphosphate, GTPase, guanosine triphosphatase, HK1, hexokinase-1, HRAS, Harvey rat sarcoma, KRAS, Kristen rat sarcoma, MAPK, mitogen-activated protein kinase, MBP, maltose binding protein, MEK, MAPK/ERK kinase, NKIRAS, NF-kappa-B inhibitor-interacting RAS-like protein, NORE1, novel RAS effector, NRAS, neuroblastoma RAS, PDZGEF, PDZ-domain-containing guanine nucleotide exchange factor, PI3K, phosphoinositide 3-kinase, PKC, protein kinase C, PLCε, phospholipase C epsilon, RA, RAS association domain, RAF, rapidly accelerated fibrosarcoma, RALA, RAS-like protein A, RALGDS, RAL guanine nucleotide dissociation stimulator, RAP, RAS proximate, RAS, rat sarcoma, RASD, dexamethasone-induced RAS-related, RASSF, RAS association domain family, RB, RAS-binding domain, RERG, RAS-related and estrogen-regulated growth inhibitor, RERGL, RAS-related and estrogen-regulated growth inhibitor-like protein, RGL, RAL guanine nucleotide dissociation stimulator-like, RGS, regulator of G protein signaling, RHEB, RAS homologous enriched in brain, RHO, RAS homologous, RIN, RAS and RAB interactor, RIT, RAS-like protein expressed in many tissues, RRAS, RAS-related protein, SARAH, Salvador–RASSF–Hippo domain, SHANK, SH3 and multiple ankyrin repeat domain, SIN1, stress-activated protein kinase-interacting protein 1, SNX17, sorting nexin-17, TIAM, T-lymphoma invasion and metastasis protein

## Abstract

RAS effectors specifically interact with GTP-bound RAS proteins to link extracellular signals to downstream signaling pathways. These interactions rely on two types of domains, called RAS-binding (RB) and RAS association (RA) domains, which share common structural characteristics. Although the molecular nature of RAS-effector interactions is well-studied for some proteins, most of the RA/RB-domain-containing proteins remain largely uncharacterized. Here, we searched through human proteome databases, extracting 41 RA domains in 39 proteins and 16 RB domains in 14 proteins, each of which can specifically select at least one of the 25 members in the RAS family. We next comprehensively investigated the sequence–structure–function relationship between different representatives of the RAS family, including HRAS, RRAS, RALA, RAP1B, RAP2A, RHEB1, and RIT1, with all members of RA domain family proteins (RASSFs) and the RB-domain-containing CRAF. The binding affinity for RAS-effector interactions, determined using fluorescence polarization, broadly ranged between high (0.3 μM) and very low (500 μM) affinities, raising interesting questions about the consequence of these variable binding affinities in the regulation of signaling events. Sequence and structural alignments pointed to two interaction hotspots in the RA/RB domains, consisting of an average of 19 RAS-binding residues. Moreover, we found novel interactions between RRAS1, RIT1, and RALA and RASSF7, RASSF9, and RASSF1, respectively, which were systematically explored in sequence–structure–property relationship analysis, and validated by mutational analysis. These data provide a set of distinct functional properties and putative biological roles that should now be investigated in the cellular context.

RAS family proteins control activities of multiple signaling pathways and consequently a wide array of cellular processes, including survival, growth, adhesion, migration, and differentiation (1). Any dysregulation of these pathways leads, thus, to cancer, developmental disorders, metabolic and cardiovascular diseases (2). Signal transduction implies a physical association of RAS proteins with a spectrum of functionally diverse downstream effectors, *e.g.*, CRAF, PI3Kα, TIAM1, RALGDS, PLCε, and RASSF5 (3, 4, 5, 6, 7, 8, 9, 10, 11). RAS-effector interaction essentially requires RAS association with membranes (12), and its activation by specific regulatory proteins (*e.g.*, guanine nucleotide exchange factors or GEFs), leading to the formation of GTP-bound, active RAS (13, 14, 15). Notably, RAS proteins change their conformation mainly at two mobile regions, designated as a switch I (residues 30–40) and switch II (residues 60–68) (16, 17, 18). Only in GTP-bound form, the switch regions of the RAS proteins provide a platform for the association of the effector proteins (19, 20).

To date, two types of domains, the RAS-binding (RB) and RAS association (RA) domains, have been defined for various effectors. They are comprised of 80 to 100 amino acids and have a similar ubiquitin-like topology (8, 21, 22, 23, 24). Considering different RAS effectors, RB and RA domain interactions with RAS proteins do not exhibit the same mode of interaction between different RAS effectors. However, CRAF RB and RALGDS RA domains share a similar structure and contact the switch I region *via* a similar binding mode (25, 26). In contrast, PI3Kα RB, RASSF5 RA, and PLCε RA domains do not share sequence and structural similarity but commonly associate with the switch regions, particularly switch I (27, 28, 29). RAS-effector interaction strikingly shares a similar binding mode adopted by three components: two antiparallel β-sheets of the RA/RB domains and the RAS switch I region, respectively, and the first α-helix of the RA/RB domains (30).

In this study, we conducted an in-depth database search in the human proteome and extracted 57 RA/RB domains. We used ten RASSF RA domains to analyze their interactions with seven representatives of the RAS proteins family, including HRAS, RRAS1, RAP1B, RAP2A, RALA, RIT1, and RHEB1. CRAF RB domain was used as control. The binding analysis was performed under the same conditions using fluorescence polarization. Obtained dissociation constants (K_d_) with a broad range (0.3–500 μM) along with a matrix for a potential interaction of 25 RAS proteins and 57 RA/RB domains provide us a detailed view of the sequence–structure–property relationships of RAS-effector binding capabilities.

## Results

### Human proteome contains 39 RA and 14 RB domain-containing proteins

Mining in the UniProt database led to the extraction of 130 RB and 145 RA-domain-containing proteins, respectively. In a parallel search using HMMER, 127 RB and 164 RA-domain-containing proteins were extracted. These numbers were reduced to 46 RB and 97 RA-domain-containing proteins by excluding proteins containing RHO-binding domains, mitochondrial proton/calcium antiporter domain, and receptors. In the last step, all isoforms with identical sequences of the RB and RA domains were excluded using multiple sequence alignments generated with the ClustalW algorithm. This approach identified a total number of 16 RB domains in 14 RB-domain-containing proteins and 41 RA domains in 39 RA-domain-containing proteins, (Fig. S1; Tables S1 and S2). Both types of RAS effector domains share sequence identity of 10.5% and 9.2% and sequence similarity of 25.5% and 20.2% (Figs. S2 and S3).

**Figure 1:**
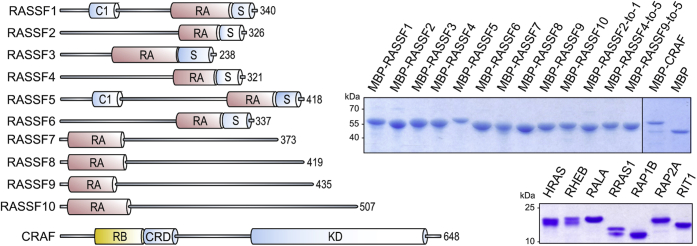
**Domain organization of effector proteins.** Schematic representation of RASSF1–10 proteins and CRAF. Different domains are highlighted, including RAS association domain (RA) in *red*, RAS-binding domain (RB) in *yellow* and other domains in *blue*. Based on their domain organization, the RASSF family proteins are divided in group 1 (RASSF1-6) and group 2 with N-terminal RA domains (RASSF7-10). Coomassie brilliant *blue* stained SDS gels show purified RAS proteins as well as the RA/RB domains purified as MBP fusion proteins.

The direct interaction of different RA-domain-containing proteins with RAS proteins has been comprehensively analyzed (23, 31). However, the majority of proteins with a RA domain remain uncharacterized (Table S1). The RAS association domain family (RASSF), which controls a broad range of signaling pathways (8, 32), is the largest RA-domain-containing protein family (Fig. 1). Their RA domains differently interact with classical RAS proteins (8, 24). Among them, only the interaction of RASSF1 and RASSF5/NORE1 RA domains has been characterized quantitatively so far (23, 31). Other characterized RA-domain-containing proteins, including RALGDS-like proteins, PLCε, AF6, RIN1/2, and PDZGEF1/2, regulate diverse cellular processes. They share high structural similarity and exhibit differential selectivity for HRAS and RAP1B (23, 31).

RB-domain-containing proteins are mostly kinases (Table S2). The serine/threonine RAF kinase family proteins (A/B/CRAF; (33)) activate the MEK-ERK axis and control cell proliferation and differentiation (34, 35). PI3Kα generates phosphatidylinositol (3,4,5)-trisphosphate (PIP_3_) and regulates cell growth, cell survival, cytoskeleton reorganization, and metabolism (36). RGS12/14, which usually act as inactivators of Gα proteins (37), physically interact with various members of the RAS family. They appear to facilitate the assembly of the components of the MAPK pathway through direct association with activated HRAS (38). TIAM1/2, which act as specific GEFs for the RHO family proteins and control cell migration (39, 40), have been suggested to recognize activated RAS proteins (41). However, their direct interaction with RAS proteins has not been shown to date (23). Moreover, a few proteins, reported as RAS effectors, do not apparently contain an RA/RB domain (Table S3).

### Variable affinities for the RAS-effector interactions

To determine the binding capability between the effector domains and diverse proteins of the RAS family, the following proteins were selected for this study: (i) all ten RASSF family proteins as representative RA-domain-containing effector proteins; (ii) CRAF RB domain (Fig. 1) was used as a representative of the RB-domain-containing proteins; and (iii) the RAS family includes 23 genes coding for at least 25 proteins, which share, considering their G domains, sequence identity of 48.6% and sequence similarity of 61.5% (Fig. S4). Based on sequence identity, structure, and function of their G domains, the RAS proteins were divided into eight paralog groups (Table S4): RAS, RRAS, RAP, RAL, RIT, RHEB, RASD, and DIRAS (42). RAS-related proteins RASLs, RERG, RERGL, NKIRAS1/2 were excluded from this list and study due to their major sequence deviations.

To monitor binding we applied a fluorescence polarization assay (21) to determine the dissociation constants (K_d_) for the RAS-effector interactions. For this, we prepared HRAS, RRAS, RAP1B, RAP2A, RALA, RIT1, and RHEB1 in complex with a nonhydrolyzable, fluorescent analog of GTP, called mGppNHp. Representatives of RASD and DIRAS groups were not applied due to their physical instability *in vitro*. Small-sized RB and RA domains were fused to maltose-binding protein (MBP, 42 kDa) to increase their overall molecular weight and to ensure a homogeneous monomeric form of the fusion proteins. Figure 1 shows SDS gels for all purified proteins used in this study.

**Figure 2:**
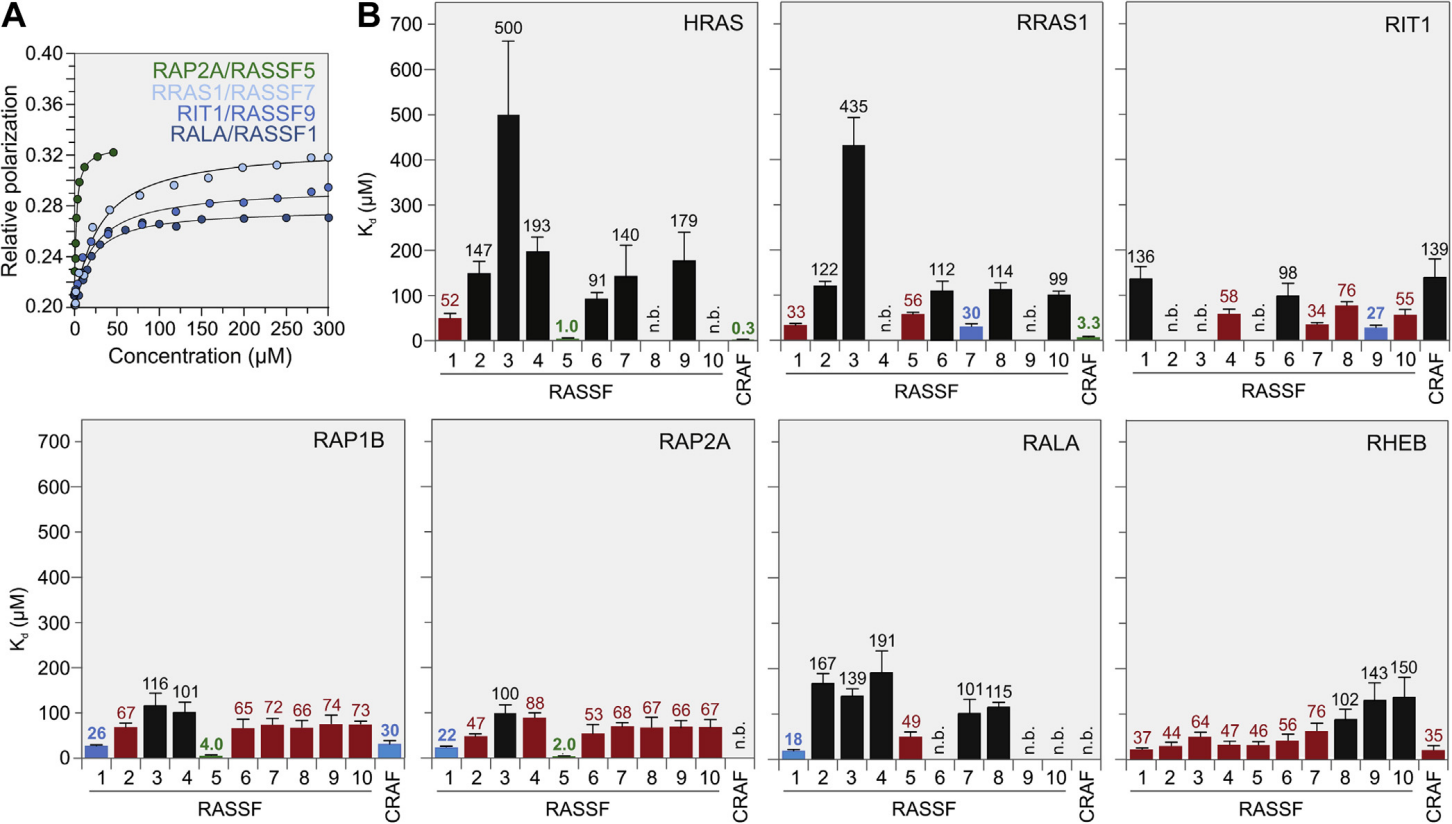
**Differential binding affinities for the RA/RB domain interactions with various RAS subfamily members.** The interactions between seven RAS subfamily members with 11 effector proteins (ten RA domains of the RASSF protein family and CRAF RB domain) were determined by titrating mGppNHp-bound, active forms of RAS proteins (1 μM, respectively) with increasing concentrations of the respective effector domains as MBP fusion proteins (Figs. S5 and S6). *A*, data of four representative experiments are shown for the interaction of RALA, RAP2A, RRAS1, and RIT1 with RASSF1, 5, 7, and 9, respectively. *B*, evaluated K_d_ values (above the bars; Table S5) were divided in high affinity (0.1–5 μM; *green*), intermediate affinity (6–30 μM; *blue*), low affinity (31–90 μM; *red*), and very low affinity (91–510 μM; *black*). No binding (n.b.) stands for K_d_ values higher than 500 μM. The error bars were derived from the fitting errors.

Increasing concentrations of MBP-fused effector proteins were titrated to RAS•mGppNHp proteins to assess the binding capability of the respective interaction pairs. We observed a significant change in fluorescence polarization for the majority of the measurements (Figs. S5 and S6*A*). However, evaluated K_d_ values ranged from 0.3 to more than 500 μM. These data are summarized in Table S5 and illustrated in Figure 2. Under these experimental conditions, the CRAF RB domain revealed the highest affinity for HRAS and RRAS1 while the RASSF5 RA domain exhibited a relatively high affinity for HRAS, RAP1B, and RAP2A (Fig. 2, *A* and *B*, green bars). The intermediate affinities were obtained for the interaction of the CRAF RB domain with RAP1B as well as RASSF1 with RAP1B, RAP2A and RALA, RASSF9 with RIT1 and RASSF7 with RRAS1 (Fig. 2, *A* and *B*; blue bars). The majority of the interaction pairs showed, however, low and very low affinities (Fig. 2*B*, red and black bars, respectively). Among them, RHEB notably revealed the majority of low-affinity interactions. No binding was observed for 12 pairwise interactions.

Purified MBP, which was titrated to HRAS•mGppNHp as a negative control, exhibited no interaction (Fig. S7*A*). The reproducibility of the fluorescence polarization measurements was assessed by determining the K_d_ value for the interaction between HRAS•mGppNHp with RASSF1-RA in three different experiments.

### Identification of common RAS-binding site pattern in RA/RB domains

**Figure 3:**
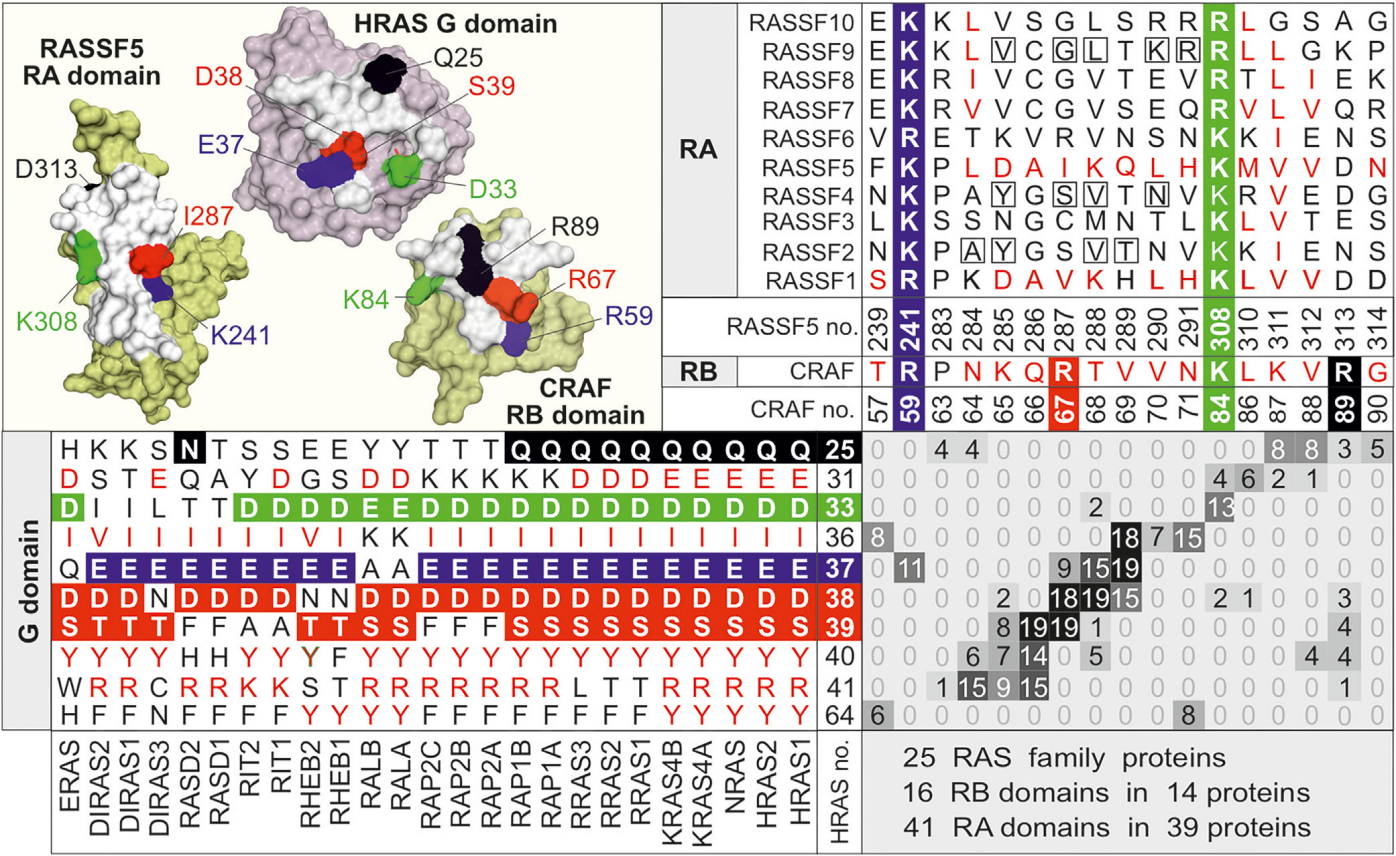
**Interaction matrix adapted for the structures of RAS complexes with effector domains.** Interaction matrix of RAS family proteins with the RA/RB proteins used in this study is generated to demonstrate interacting residues in respective structures (see Table S6). It comprises the amino acid sequence alignments of the RAS proteins (*lower left panel*) and the effector domains (*upper right panel*), respectively, extracted from the complete alignments in Figs. S2–S4. Each element corresponds to a possible interaction of RAS residues (*row*; HRAS numbering) and effector (*column*; CRAF and RASSF5 numbering, respectively). The number of actual contact sites between RAS and the effector domains (with distances of 4 Å or less) were calculated and are indicated with positive numbers for matrix elements. Extracted structures of HRAS (in orchid) and the RA domain of RASSF5 and RB domain of CRAF (in olive) from their surface complexes are presented (*top left panel*). Key interaction hotspots with the same color codes are highlighted on the surface structures as well as in the interaction matrix and the secondary structures, respectively. Boxed residues in RASSF2, 4, and 9 were replaces to RASSF1 and 5, respectively, to validate the impact of these hotspot residues on the interaction with RAS family proteins (Fig. 4).

To understand the atomic interactions between RAS and effector proteins and explain observed variable affinities, we analyzed various structures of RAS-effector protein complexes. To date, 13 structures of RAS-effector protein complexes exist in the PDB (Table S6). As some of them contain more than one complex in the unit cell, there were altogether 19 complexes available for the analysis. In order to map atomic interactions responsible for observed variable affinities, we have extracted information about interacting interface from all of the abovementioned complex structures (Figs. S8 and S9) and combined them with their sequence alignments (Figs. S2–S4). It is important to note that some amino acids, aligned according to the sequence, were quite distant in the space. Therefore, we edited the sequence alignment to synchronize it with the structural alignment. Our python code finally took sequence alignments with PDB files of complex structures as inputs and calculated all interaction pairs in analyzed complex structures in the form of an interaction matrix. The resultant matrix comprehensively relates the interacting residues on both sides of the complexes, with RAS paralogs as rows and the RA/RB domains as columns (Fig. 3). All numbering in this study is based on HRAS on the one side and CRAF and RASSF5, for RB and RA domains respectively, on the other side.

Each element of the matrix that can be accounted for a “hotspot” relates one homologous residue from RAS proteins to one homologous residue from the RA/RB domains. The number value of this element, ranging from 0 to 19, represents the number of complex structures in which these residues interact (Fig. 3). Thus, 0 means that these two residues do not contact each other in any structure while a maximal value 19 means that this particular interaction exists in all analyzed complex structures of the RAS-RA/RB domains. We have sorted the residues at both sides of the matrix according to their conservation *versus* variability. As can be seen in Figs. S4 and S9, the majority of the residues (14 out of 20) on the side of 25 RAS proteins are conserved, nine of which (Q/N25, D/E33, I/V36, E37, D38, S/T39, Y40, R/K41 in switch I, and Y64 in the switch II; HRAS numbering) account for major hotspots (Fig. 3). On the other side, and in contrast, the majority of 19 RAS interacting residues in RA/RB domains are variable and only two distant residues are conserved (R/K59 and K/R84; CRAF numbering; R/K241 and K/R308; RASSF5 numbering) (Fig. 3 and Fig. S9).

However, what is striking is the middle cluster of the matrix with the most frequent interactions between the conserved residues in the switch I region of the RAS proteins (β2-strand residues 36–41; HRAS numbering) and the variable residues of the RA/RB domains (β2-strand residues 64–71; CRAF numbering; residues 284–291; RASSF5 numbering) (Fig. 3 and Fig. S9). This cluster adopts an arrangement of intermolecular β-sheet interactions in an antiparallel fashion (Fig. S8). A substantial number of the contacts in this cluster are mediated by main-chain/main-chain interactions, which typically involve hydrogen bonds between the N-H group and the carbonyl oxygen of the amino acids 37 to 39 from the RAS side and positions 66 to 69 (CRAF numbering) and 286 to 289 (RASSF5 numbering) from the side of the RA/RB domains.

### Switched RASSF-binding selectivity by hotspots residue swapping

To prove the impact of the hotspot residues on the selectivity of the RASSF RA domain interactions with RAS family proteins, we selected the weak and strong RAS-RASSF interactions, and substituted 4 to 5 amino acids in the hotspot region (Fig. 3, boxed residues) RASSF2 to RASSF1 as well as RASSF4 and RASSF9 to RASSF5. The variants, RASSF2-to-1, RASSF4-to-5, and RASSF9-to-5 (Fig. 1), were successfully expressed and purified. Their binding affinities for HRAS, RIT1, RALA, RAP2A and RRAS1 were measured using fluorescence polarization (Fig. S10).

**Figure 4:**
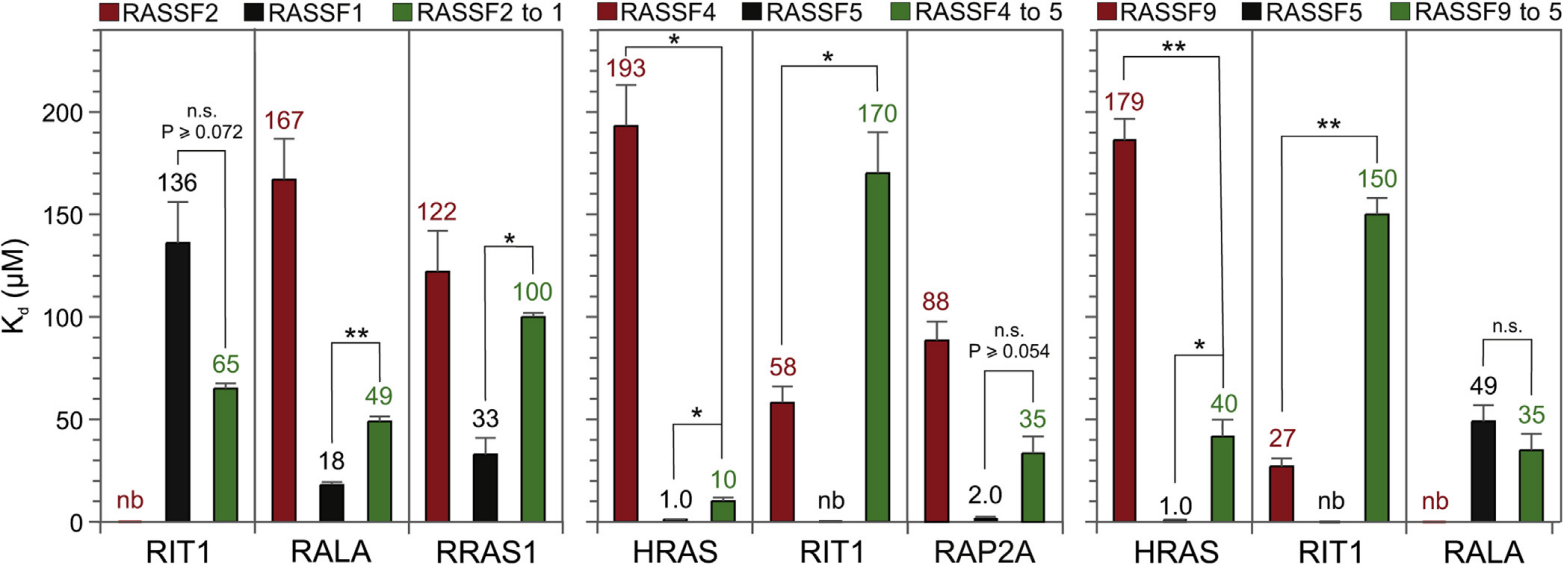
**Validation of RAS-RASSF selectivity by hotspots residue swapping in RASSF RA domains.** The interactions of the RASSF hotspot variants (RASSF2-to-1: A186K/Y187D/V190K/T191H, RASSF4-to-5: Y185D/S187I/V188K/N188L and RASSF9-to-5: V40D/G42I/L43K/K45L/R46H; see Fig. 3, boxed residues) with various RAS family proteins (RIT1, RALA, RRAS1, HRAS, and RAP2A) were determined by fluorescence polarization (see Fig. S10), and evaluated K_d_ values were plotted as bar charts together with K_d_ values of wild-type RASSF1, 2, 4, 5, and 9. (∗*p* < 0.05; ∗∗*p* < 0.01). Color codes highlight RASSF wild types (*red* and *black*) and RASSF variants (*green*). The error bars were derived from the fitting errors.

Remarkable differences in binding affinities of the analyzed RASSF variants are summarized in Figure 4 for comparison. RASSF2-to-1 variant revealed a significant increase of RALA (*p* < 0.006) and RRAS1 (*p* < 0.014) binding affinity compared with RASSF2 but declined compared with RASSF1. In contrast, RIT1, which did not show any binding to RASSF2 and a very low affinity to RASSF1, now exhibited a reasonable K_d_ value of 65 μM for RASSF2-to-1. The RASSF4-to-5 variant, on the one hand, showed a tremendous increase in affinity for HRAS of about 20-fold (*p* < 0.0118) and, on the other hand, diminished RIT1 property to bind RASSF4 by threefold (*p* < 0.0351). These data suggest that the hotspot residues favor RASSF4 binding to RIT1, whereas those residues of RASSF5 counteract RIT1 binding. Similarly, the RASSF4-to-5 affinity for RAP2A was increased by 2.5-fold (n.s., *p* < 0.087), which emphasizes the high-affinity RAP2A-RASSF5 interaction. The RASSF9-to-5 variant showed a 4.5-fold increase in HRAS-binding affinity as compared with RASSF9 (*p* < 0.008) that can be attributed to the high-affinity interaction of HRAS with RASSF5. The intermediate affinity of RASSF9 for RIT1 of 27 μM is validated by the RASSF9-to-5 variant, which revealed a 5.5-fold higher K_d_ value (*p* < 0.005). The interaction of the RASSF9-to-5 variant with RALA was drastically enhanced (K_d_ = 35 μM) considering the lack of RASSF9 binding to RALA.

Our data on residue swapping in RASSF proteins successfully validated the key role of hotspot residues in the RAS-RASSF interaction, particularly RASSF1-RALA, RASSF5-HRAS, and RASSF9-RIT1.

### RIT1 pull-down from cell lysates by RASSF7 and RASSF9

**Figure 5:**
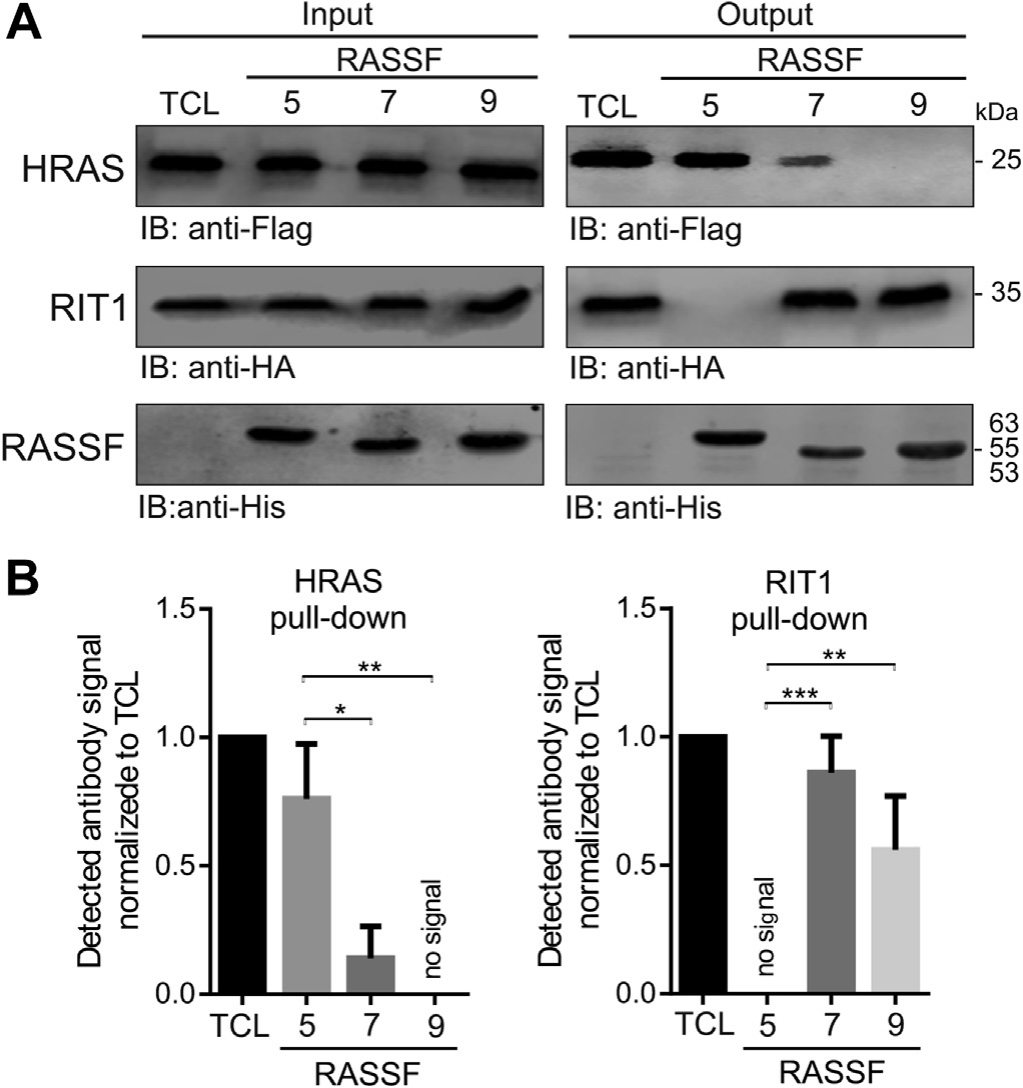
**Binding analysis of RIT1 and HRAS with RASSF1-RA, RASSF7-RA, and RASSF9-RA using pull-down assay.***A*, HA-RIT1 and FLAG-HRAS, overexpressed in HEK 293T cells, were pulled down using the His-tagged MBP-RA domains of RASSF5, RASSF7, and RASSF9, respectively, and immunoblotted (IB) using anti-HA and anti-FLAG antibodies. Immunoblots of total cell lysates (TCL) were served as a loading control to detect HA-RIT1 and FLAG-HRAS. An anti-His antibody was used for detection of the His-tagged MBP-RA domains of RASSF5, RASSF7, and RASSF9 as an input loading control (more detail in Fig. S11). *B*, the graphs represent densitometric analysis of three independent experiments under the same conditions as shown in (*A*). All values were normalized to the loading control. All data are expressed as the mean of triplicate experiments ± standard deviation (unpaired *t*-test, ∗*p* < 0.05, ∗∗*p* < 0.01, and ∗∗∗*p* < 0.001).

To prove physiological relevance of identified RIT1 interactions with RASSF7 and RASSF9, we transfect Human Embryonic Kidney (HEK) 293T cells with human RIT1 and used His-tagged RA domains of RASSF7 and RASSF9 to pull down HA-tagged RIT1 from the cell lysates. As a control, we used lysates of HRAS-transfected cells and His-tagged RASSF5 RA domain to pull down FLAG-HRAS. As shown in Figure 5*A*, RIT1 bound to RASSF7 and RASSF9 but not to RASSF5, which was, in contrast, able to pull down HRAS. Data of three independent experiments were quantified by a Li-Cor Odyssey imaging system and expressed as signal intensity (Fig. 5*B*), confirmed the significance of the RIT1 interaction with RASSF7 and RASSF9 (*p* < 0.01).

## Discussion

Effector selection and activation by a RAS protein in a proper cellular context and appropriate protein network are known to initiate a cascade of biochemical reactions and thus control defined cellular functions in all types of cells. It is also increasingly clear that functionalization of the effectors with various modular building blocks, mainly the RA/RB domains, is a prerequisite for successful orchestration of a series of spatiotemporal events, including recruitment, subcellular localization, assembly of proactive protein complexes, and ultimately association with and activation *via* the RAS protein. An issue that is investigated in-depth in this study is how many effectors for RAS proteins exist in the human proteome and how they achieve the desired affinity and selectivity for their cognate RAS protein.

The total numbers of RAS effectors differ from study to study. A SMART database search has provided 108 RA and 20 RB-domain-containing proteins in one of the early and first comprehensive studies on RAS-effector interactions (23). These numbers have been slightly reduced to 100 RA domains and only a few members of RB-domain-containing proteins, including A/B/CRAF, TIAM1/2, and RGS12/14 proteins (31). In the next studies, Kiel *et al.* (43) have come to around 70 human proteins, containing RA and RB domains. Ibáňez Gaspar *et al.* (44) have analyzed in their very recent, comprehensive study 56 established and predicted RAS effectors with the potential ability to bind to RAS oncoproteins. Our search, using the UniProt database and the program HMMER, alongside with a cross-check of each individual sequence, ended up with 41 RA in 39 RA-domain-containing proteins and 16 RB in 14 RB-domain-containing proteins (Fig. S1). Thus, our lists contain 53 proteins, also including RALGDSL2 and SNX17 (Tables S1 and S2). SNX17 along with SNX27 and SNX31, which possess a FERM-like domain, has been shown to directly bind to GTP-bound HRAS (45) and may thus be involved in endosomal RAS signaling processes (46). However, we exclude RASGEF3-5, KRIT1, and RGL4. Sequences, related to RA or RB domains, were not found in other proteins (Table S3), such as SIN1, SNX31, HK1 (Hexokinase 1), and SHANK2-3, which have been recently described as new RAS effector proteins (45, 47, 48, 49, 50).

In order to refine a comprehensive list of RAS proteins and their effectors regarding their capabilities of mutual binding, we have investigated pairwise interaction between selected proteins (Fig. 2), related them to available structural data (Fig. 3), and combined them with data described in previous studies (Fig. S9).

The RASSF family contains ten members and is divided into two groups; RASSF1–6 typically have C-terminal RA and SARAH domains and RASSF7–10 an N-terminal RA domain (Fig. 1) (51). However, RAS-binding residues are not conserved in group 2 of the RASSF family and overall, the RA domains of these two RASSF groups are about 25% identical. Our data showed a much lower binding affinity between RAS family members and RA domains of group 2 (Fig. 2).

In a very recent study, Dhanaraman *et al.* have performed RASSF pull-down experiments under similar conditions as previously published by Chan *et al.* (8, 24). As already stated by the reviewer, this approach has limitations to detect affinities lower than 10 to 30 μM, which is dependent on several variables such as the buffer and centrifugation speed. Chan *et al.* have observed HRAS interactions with RASSF6 and RASSF7 as well, which were determined in the present study, although with very high K_d_ values (Fig. 2). RASSF5 binding to KRAS and HRAS, as reported by Dhanaraman *et al.*, also confirms a previous study by Nakhaeizadeh *et al.*, which has shown similar binding affinities of the effector domains, including RASSF5-RA, toward the RAS paralogs, HRAS, KRAS, and NRAS (21). In contrast, Dhanaraman *et al.* have examined RAP1A and RASSF5, consistent with our study with RAP1B and RAP2A (Fig. 2), but did not consider them as interactions, again due to the approach limitation of 10 μM (see below).

RASSF1 and RASSF5 RA domains share the highest sequence homology and several residues, including L282, D285, A286, I/V287, K288, H291, K308, V311, V312, and D313 (RASSF5 numbering), involved in RAS interaction (Fig. 3), are almost identical. These RASSFs have been described in many studies as effectors for H/K/NRAS, RRAS1, and RAP1A (19, 32, 52, 53). Accordingly, we have determined high and intermediate affinities for their association with RAS family members in this study (Fig. 2) and in part also in a previous report (21). Interestingly, RASSF proteins turned out to interact with several other RAS-related proteins, beyond the classical RAS paralogs. Shifman and colleagues have recently shown by immunoprecipitation experiments that RASSF1 also interacts with ERAS and DIRAS3 (54), which are atypical members of the RAS family (42). Similarly, Dhanaraman *et al.* (24) have very recently demonstrated the interaction of RASSF1 with GEM, REM1, REM2 and RASL12 GTPase proteins. These GTPases, which belong to the RGK GTPase family, regulate voltage-dependent calcium channels and cell shape (24). The present study showed that RIT1 interacts RASSF7 and RASSF9, and RALA with RASSF1. These interactions, which successfully validated the key role of hotspot residues in the RAS-RASSF interaction (Fig. 4), confirmed our predicted interaction model (Fig. 3). The RALA-RASSF1 interaction seems rather relevant since the presence of four RASSF1 hotspot residues in RASSF2 considerably enhanced RALA binding (Fig. 4). RALA and RALB contain lysine and alanine at positions 36 and 37, respectively (HRAS numbering), rather different residues than isoleucine and glutamate in other RAS proteins, which are known to be critical for the RAS-effector interactions (55). RALA-RASSF1 interaction has not been reported to date and awaits further cell-based investigations, especially because the RASSF2-to-1 variant gained binding affinity toward RALA (Fig. 4). Similarly, RALA-RASSF5 interaction appears rather relevant as the RASSF9-to-5 variant affected the binding of RALA. This has been also demonstrated by hotspots residue swapping of RASSF9 to RASSF5. While RALA showed a K_d_ value of 35 μM for RASSF9-to-5 while it did not show any binding to RASSF9, suggesting that very few key residues are sufficient to generate the appropriate binding surface. This notion presumes that analyzed RA domains share a conserved mode of RAS recognition based on the formation of an intermolecular, antiparallel β sheet (21, 24).

Among all RASSF family members, only RASSF1 and RASSF5 interact in high or intermediate affinities with all investigated RAS family members, with an exception of RIT1 (Fig. 2). RASSF7-9 RA domains share high sequence similarity and are different from RASSF10 (Fig. S2). A common signature of the RASSF members is the existence of the K/R241 and K/R308 hotspots (Fig. 3). They revealed, with a few exceptions, comparable K_d_ values for different representatives of the RAS family (Fig. 2). RIT1-RASSF7 and RIT1-RASSF9 interactions with affinities of 34 and 27 μM are quite remarkable, especially because these proteins have not been reported yet as RAS effectors. RIT1 contains an alanine instead of the conserved S/T39 (HRAS numbering), and RASSF9 contains two negatively charged glutamic acids instead of the positively charged lysine residues at 307 and 308 (RASSF5 numbering; Fig. S2). These two drastic deviations may be responsible for the very low affinity of RASSF9 for HRAS due to electrostatic repulsion with D33. However, RIT1 contains also an aspartic acid at the corresponding position and yet shows an intermediate affinity for RASSF9. The relevance of RIT1-RASSF9 interaction was successfully validated by residue swapping. Substitution of five RASSF9 to RASSF5 residues, which did not bind RIT1, significantly impaired the interaction (Fig. 4). Moreover, cell-based pulldown experiments confirmed the relevance of RASSF7 and RASSF9 as potential RIT1 effectors and support the notion that K_d_ values of about 30 μM can be considered physiologically relevant. It is important to note that GTPase-effector interactions in the cell take place in a context of multivalent platform very different from the isolated domains and bimolecular interaction under cell-free conditions. Effectors are full-length, associated with accessory proteins, and eventually the cell membrane, and are subjected to distinct control mechanisms, including posttranslational modifications. Validated antibodies against these proteins will enable us in near future to take the next step namely determining the appropriate cell type that endogenously expresses the desired proteins and to unambiguously verify RIT1-RASSF interaction.

RHEB broadly exhibited low-affinity interaction with RASSF1-7, particularly RASSF1 (Fig. 2), which may be based on a large number of amino acid deviations in both switch regions (Fig. 3 and Fig. S4). It has been proposed that RHEB may complex with RASSF1 to coordinate signaling pathways, after processing by MST/LATS and TOR kinases (56). In the presence of RASSF1, RHEB has been shown to stimulate the MST/LATS/YAP pathways but is suppressed in its ability to activate the TOR pathway. The physical interaction of RHEB with RASSFs remains to be shown in cells, like it has been shown for other RAS and RAS-like proteins (54).

CRAF RB domain is one of the most and best studied RAS effectors with the highest selectivity for the H/K/NRAS paralogs and to a certain extent also for the RRAS proteins (21). CRAF RB domain revealed an intermediate affinity for RAP1B and RHEB1 but not for RIT1 or RAP2A (Fig. 2). The RAP1 and RAP2 subgroups differ at positions 25 and 39 (HRAS numbering), which are in the case of RAP1 proteins occupied by favorable glutamine and serine (Fig. 3). The two orders of magnitude lower affinity of RAP1B for the CRAF RB domain stems from the drastic deviation at position 31 (HRAS numbering). K31 in RAP proteins obviously collides with the K84 in CRAF and disfavors a RAP-CRAF interaction (Fig. S9); this was the reason why RAP1A mutated at this site was used for successful determination of the complex structure between RAP1A and the CRAF RB domain (26). Phosphorylation of RAP1A at S11 has been recently proposed to promote RAP1A-CRAF RB domain interaction (57). Devanand and colleagues have proposed that phosphorylation of S11 allosterically modulates the dynamics of RAP1A switch regions, which consequently promotes the RAP1A-CRAF complex formation and downstream signaling (58).

An intermediate affinity for CRAF RB domain interaction with RHEB G domain (Fig. 2) points to previous reports of a direct relationship between these two crucial signaling molecules. PKA-dependent phosphorylation of CRAF at S43 has been shown to reciprocally potentiate RHEB-CRAF interaction and to decrease CRAF interaction with HRAS (59). An asparagine instead of D38 (HRAS numbering) in the switch I region seems to be critical for the unique CRAF-binding properties of RHEB. In a different study, Henske and coworkers have shown that RHEB interacts with and inhibits BRAF (60). In this context, RHEB not only hinders the BRAF association with HRAS but also interferes with BRAF activation and its heterodimerization with CRAF. As the RB domains of the RAF paralogs are conserved (33), mainly regarding their RAS-binding residues (Fig. S3), differences between BRAF and CRAF interactions with RHEB may stem from deviations outside the RB domains or from different phosphorylation states. Heard *et al.* (61) have recently reported a strong interaction between RHEB-GTP and BRAF (but not with CRAF) and that RHEB overexpression decreases and RHEB knockdown increases RAF/MEK/ERK activation. They have shown that a variant of RHEB (Y35 to asparagine; Y32 in HRAS) impedes RHEB interaction with BRAF leading to an increased BRAF/CRAF heterodimerization and thus activation of the MAPK pathway. Accordingly, they have proposed a dual function for RHEB, suppression of the MAPK pathway and mTORC1 activation (61).

RIT1-CRAF interaction has been frequently proposed due to their critical roles in developmental disorders, collectively called RASopathy (62), but not directly shown. We observed a very low affinity for these two proteins (Fig. 2), which may stem from the sequence deviation between RIT1 and HRAS in their switch I region (Fig. 3). In an early study on biochemical characterization of RIT, Andres and coworkers have shown that RIT1 interacts with RA domains of RALGDS and AF6 but not with the CRAF RB domain (63). In a different study, they have shown that RIT1 binds and activates BRAF but not CRAF (64). This may again implicate those additional regions may exist outside the conserved RB domains of the RAF paralogs, which differently facilitate the interaction with the RAS proteins, such as RIT1 or RHEB.

An ever-present central concern in the biophysical investigation of protein–protein interactions is the relevance of low (10–30 μM) to very low (>> 30 μM) affinity interactions in the regulation of signaling events. These protein complexes rely on weak, transient interactions that are emerging as important components of large signaling complexes at the plasma membrane that are required to respond to external stimuli. Cellular membranes play a critical role in the localization and orientation of protein complexes and in fine-tuning of protein functions (65). The activity of RAS and RAF paralogs is regulated through different parameters, including membrane association. Analysis of dynamic interactions between KRAS4B and lipid bilayer membrane has revealed that association of ARAF RB domain with active KRAS4B not only reorients KRAS4B at the membrane surface but also facilitates membrane binding of ARAF RBD itself (66). Four basic residues, K28, K66, R68, and K69, are engaged in lipid binding. Another emerging concept is based on the physical interaction of the G domain itself with a lipid membrane. A membrane-based, nucleotide-dependent conformational switch operates through distinct regions on the surface of RAS proteins, including the hypervariable region (HVR), which reorients with respect to the plasma membrane (67, 68, 69, 70, 71, 72, 73, 74, 75, 76). Mazhab-Jafari and colleagues have proposed two different orientations of KRAS4B facing toward the membrane (66). KRAS4B in an exposed GDP-bound form favors α4/α5 helices, which considerably reorients upon activation, and favors β1-β3 sheets and α2/α3 helices from the G domain, and K167/K172 from HVR, in an occluded GTP-bound form. G-domain-membrane interaction may not only stabilize protein complexes but may also contribute to the specificity of signal transduction. A critical aspect in this context is the organization of RAS proteins into protein–lipid complexes. These so-called nanoclusters concentrate RAS at the plasma membrane. They are the sites of effector recruitment and activation and are essential for signal transmission (67, 70, 77, 78).

A frequently encountered issue in the enhancement of RAS-effector interaction is posttranslational modification. Thurman *et al.* (79) have recently demonstrated that the ubiquitylation of KRAS at L147 impairs RAS-RASGAP interaction and facilitates RAS-CRAF association and MAPK signaling. Barceló *et al.* (80) have shown that PKC-catalyzed phosphorylation of KRAS at S181 results in an increased interaction of KRAS with CRAF and PI3Kα. Several studies have previously shown that the CRAF CR domain undergoes direct interaction with HRAS, which appears to be enhanced by the farnesyl moiety if using farnesylated RAS (15, 81, 82, 83, 84, 85, 86). A possible HRAS-CRAF CR domain interaction has been proposed to be, contrary to the CRAF RB domain, outside of the switch regions of HRAS and thus independent of its nucleotide-bound state. In contrast, Y32 and Y64 phosphorylation by SRC alters the conformation of switch I and II regions, markedly reduces RAS binding to CRAF, and concomitantly increases binding to RASGAPs and the rate of GTP hydrolysis (87, 88).

Another aspect related to very low affinity interactions involves a secondary RAS-binding site, in addition to the RA/RB domain, in terms of a two-step, two-domain binding model. The two-domain model accommodates at least two different enhancer mechanisms. One is the direct enhancement of a selective RAS-effector interaction required for effector activation, proposed for the interactions of yeast RAS2 with two sites in adenylyl cyclase (89), HRAS with RB and CR domains of CRAF (33), and HRAS with two RA domains of PLCε (90). The latter may involve a high-affinity, GTP-dependent binding of the RA2 domain accompanied by low-affinity, GTP-independent binding of the RA1 domain. The deletion of one of the RA domains inhibits HRAS-induced PLCε activation (90). Notably, AF6 also possesses two RA domains and two RGS12/14 RB domains, respectively (44). Such a tandem arrangement of RA with RB domains may enhance their affinity toward RAS, increase effector occupancy by additional endogenous events and thus the signaling output. An emerging concept, therefore, is the action of membrane-binding CR domain that stabilizes RAS-CRAF RB domain interaction accompanied by S621 phosphorylation and 14-3-3 binding that collectively facilitates RAF activation (82, 83, 91, 92, 93, 94).

The formation of multiprotein complexes underlies a multistep assembly mechanism that follows a defined and probably short path from the cytoplasm, just underneath the membrane, to the membrane where membrane-associated proteins, for example, RAS proteins, are anchored. The first step, which has been designated as the piggyback mechanism (95), most likely increases local concentrations of protein components in a small volume and may drive cytoplasmic phase separations (96, 97, 98). The second step is the site-specific association of assembled protein complex with membrane-associated components, such as RAS proteins, which in turn are connected to receptors and coreceptors (44, 97, 98). In this way, a machinery of signaling molecules is orchestrated before the ligand activates the receptor. This is fine-tuned and prepared for an efficient signal transduction. Of course, it remains to be figured out why some interactions are in the nanomolar range (*e.g.*, 20 nM) and some in the micromolar range (*e.g.*, 20 μM or more). Given that the latter is involved in the initiation of multivalent macromolecular interactions, the final complex formation comes along after multivalent interactions have proceeded (99). This obviously increases significantly both the number of interacting complexes and overall binding affinity by orders of magnitude (44). The nanomolar affinity, however, may determine the selectivity for a sequential formation of two complexes. These interactions are often characterized by fast association and slow dissociation rates, indicating the formation of stable complexes (100, 101, 102).

## Experimental procedures

### Constructs

Gene fragments encoding RAs of RASSF1 (accession number Q9NS23; amino acids or aa 194–288), RASSF2 (P50749; aa 176–264), RASSF3 (Q86WH2; aa 79–187), RASSF4 (Q9H2L5; aa 174–262), RASSF5 (Q5EBH1; aa 200–358), RASSF6 (Q6ZTQ3; aa 218–306), RASSF7 (Q02833; aa 6–89), RASSF8 (Q8NHQ8, aa 1–82), RASSF9 (O75901, aa 25–119), and RASSF10 (A6NK89; aa 4–133) as well as CRAF RB domain (P04049, aa 51–131) were cloned into pMal-c5X-His vector. The variants RASSF2-to-1 (A186K/Y187D/V190K/T191H), RASSF4-to-5 (Y185D/S187I/V188K/N188L) and RASSF9-to-5 (V40D/G42I/L43K/K45L/R46H) were generated by BioCat Gene Synthesis (BioCat GmbH) in pMal-c5X-His vector. Constructs for the prokaryotic expression of human HRAS, RRAS, RALA, RHEB1, RIT1, RAP2A, and RAP1B isoforms were described previously (6). For mammalian expression, human HRAS and RIT1 were cloned in pcDNA3.1-Flag and pMT2-HA vectors, respectively.

### Proteins

All RASSF and RAS proteins were expressed in *Escherichia coli* using the pMal-His and pGEX expression systems and purified using Ni-NTA and glutathione-based affinity chromatography as described previously (103). RAS•mGppNHp was prepared as described (103). mGppNHp is a fluorescent, nonhydrolyzable analog of GTP; m stands for the methylanthraniloyl (m) and GppNHp for Guanosine-5'-[(β,γ)-imido]triphosphate.

### Fluorescence polarization

Increasing concentrations of RA/RB domains (0.002–300 μM) were added to the solution of mGppNHp-bound RAS family proteins (1 μM) in a buffer, containing (30 mM Tris-HCl, pH 7.4, 100 mM NaCl, 5 mM MgCl_2_, 3 mM DTT) using fluorescence polarization on a Fluoromax 4 fluorimeter as described previously (75). The excitation wavelength was 360 nm and the emission wavelength 450 nm. The dissociation constants (K_d_) for the RAS-effector interaction were evaluated using a quadratic ligand-binding equation.

### Bioinformatics

Information about RB and RA domains was obtained either from annotations in the UniProt database or in parallel using the program suite HMMER [http://hmmer.org/]. HMMER uses a Hidden Markov Model to compare sequences. Unlike CLUSTAL, which directly compares corresponding amino acids in the alignment, HMMER also takes adjacent amino acids into account. To do so, it calculates a Profile HMM before sequence comparison. It determines which amino acids are suitable at a given position. In the context of some protein domain, Profile can be viewed as a mapping of its characteristic features required for the domain structure, function, or interaction. Sequence alignments were performed in the Bioedit program using the ClustalW algorithm (104). By using Chimera, the sequence alignments were adjusted with superimposed structures (25). An interaction matrix is based on intermolecular contacts in complex structures (21). A python code was written to match sequence alignments with complex structures and calculated intermolecular contacts were put in the form of the interaction matrix. The intermolecular contacts were defined as pair residues with a distance of 4.0 Å between effectors and RAS proteins in available complex structures in the protein data bank (http://www.pdb.org). Biopython modules (105) were also used to elucidate corresponding residues in all available complex structures. The structural representation was generated using Pymol viewer (http://www.pymol.org).

### Cell-based assays

In total, 3.2 millions of HEK 293T cells were seeded in 10 cm plates in DMEM supplemented with 10% fetal bovine serum (FBS) 14 h prior to transfection. The cells were transfected at 80% to 90% confluency using TurboFect transfection reagent (R0532, Thermo Scientific), with Flag-tagged HRAS and HA-tagged RIT1 constructs, or no plasmid as a negative control. At 24 h posttransfection, cells were washed in ice-cold phosphate-buffered saline (PBS) and lysed in ice-cold lysis buffer, containing 50 mM Tris/HCl pH 7.5, 5 mM MgCl_2_, 100 mM NaCl, 1% Igepal CA-630, 10% glycerol, 20 mM ß-glycerolphosphate, 1 mM Na-orthovanadate, EDTA-free inhibitor cocktail 1 tablet/50 ml. In total, 200 μg cell lysate was added to 20 μg His-tagged MBP-RASSF proteins coupled with 100 μl Ni-NTA beads. The samples were incubated for 30 min on the rotator at 4 °C. After three washing with the lysis buffer and centrifugation steps (30 s at 300*g*), the samples were subjected to SDS-PAGE (12.5% polyacrylamide). HRAS and RIT1 were detected by immunoblotting using a rabbit anti-His (RM146) antibody (Thermo Fisher), a rabbit polyclonal anti-FLAG (F7425) antibody (Sigma), and a rabbit polyclonal anti-HA (SC-805) antibody (Santa Cruz), respectively. The immunoblots were evaluated using an Odyssey Fc Imaging System (LI-CORE Biosciences).

## Data availability

All the data are in the article.

## Supporting information

This article contains supporting information.

## Conflict of interest

The authors declare that they have no conflicts of interest with the contents of this article.
