# Supplementary material for: A comprehensive analysis of RAS-effector interactions reveals interaction hotspots and new binding partners
Source: J Biol Chem. 2021 Apr 28;296:100626. doi: 10.1016/j.jbc.2021.100626 (PMC8163975; doi:10.1016/j.jbc.2021.100626)
Supplement: Supplemental Figures S1–S11 and Tables S1–S6 [file mmc1.pdf]

Supporting Information

A comprehensive analysis of RAS-effector interactions reveals interaction hotspots and new binding partners

Soheila Rezaei Adariani, Neda S. Kazemein Jasemi, Farhad Bazgir, Christoph Wittich, Ehsan Amin, Claus A. M. Seidel, Radovan Dvorsky, Mohammad Reza Ahmadian
Institute of Biochemistry and Molecular Biology II, Heinrich Heine University, Medical Faculty, 40225 Düsseldorf, Germany

Table S1. Human proteins containing RAS association (RA) domain

No.	Entry	Protein name
1	Q9NS23	RASSF1, NORE2, PDA32
2	P50749	RASSF2, CENP34, RASFADIN
3	Q86WH2	RASSF3
4	Q9H2L5	RASSF4
5	Q8WWW0	RASSF5, RAPL, NORE1
6	Q6ZTQ3	RASSF6
7	Q02833	RASSF7, HRC1
8	Q8NHQ8	RASSF8, HOJ1
9	O75901	RASSF9, PCIP1, PAMCI
10	A6NK89	RASSF10
11	P55196	AF6, AFDN, MLLT4
12	Q12967	RALGDS, RALGEF, RGF, RGDS
13	O15211	RALGDSL2, RAB2L
14	Q9NZL6	RGL1
15	Q9BSI0	RGL2
16	Q3MIN7	RGL3
17	Q9Y4G8	PDZGEF1, RAPGEF2, RAGEF1
18	Q8TEU7	PDZGEF2, RAPGEF6, RAGEF2
19	Q9P212	PLCε1, PPLC, NPHS3
20	Q13671	RIN1, JC99
21	Q8WYP3	RIN2, JC265
22	Q8TB24	RIN3
23	Q5U651	RAIN, RASIP1
24	Q7Z5R6	RIAM, APBB1IP, PREL1, RARP1
25	Q96JH8	RADIL, RASIP2
26	Q14451	GRB7, B47
27	Q13322	GRB10, GRB-IR, Meg1, RSS
28	Q14449	GRB14
29	Q15036	SNX17
30	Q96L92	SNX27
31	Q70E73	RAPH1, PREL2
32	P52824	DGKQ
33	Q96P48	ARAP1
34	Q8WZ64	ARAP2
35	Q8WWN8	ARAP3
36	B2RTY4	MYO9A
37	Q13459	MYO9B
38	Q9HD67	MYO10
39	Q9P2F6	ARHGAP20

Table S2. Human proteins containing RAS binding (RB) domain

No.	Entry	Protein name
1	P10398	ARAF, RAFA1, PKS
2	P15056	BRAF, NS7, p94
3	P04049	CRAF, CMD1NN, NS5
4	P42336	PI3Kα, p110α, CLAPO, CLOVE
5	P42338	PI3Kβ, p110β
6	P48736	PI3Kγ, ß110γ, PIK3
7	O00329	PI3Kδ, p110δ
8	O00443	PI3KC2A, PI3KC2α
9	O00750	PI3KC2B, PI3KC2β
10	O75747	PI3KC2G, PI3KC2γ
11	O14924	RGS12
12	O43566	RGS14
13	Q13009	TIAM1
14	Q8IVF5	TIAM2, STEF

Table S3. Proposed RAS effectors with no RA/RB domains

No.	Entry	Protein name	Reference
1	Q8IZJ4	RGL4	[1]
2	O95398	RAPGEF3, Epac1	[1, 2]
3	Q8WZA2	RAPGEF4, Epac2	[1, 3]
4	Q92565	RAPGEF5, Repac	[1]
5	O00522	KRIT1, Krit	[1, 4]
6	P19367	HK1	[5]
7	Q9BPZ7	SIN1, MAPKAP1	[6]
8	Q9BYB0	SHANK3	[7, 8]
9	Q9UPX8	SHANK2	[8]
10	Q8N9S9	SNX31	[9]
11	Q75LH2	FLJ10324	[10]

[1] Ibáňez Gaspar V, Catozzi S, Ternet C, Luthert PJ, Kiel C. Analysis of Ras-effector interaction competition in large intestine and colorectal cancer context. Small GTPases. 2020:1-17.
[2] Kiel C, Wohlgemuth S, Rousseau F, Schymkowitz J, Ferkinghoff-Borg J, Wittinghofer F, et al. Recognizing and defining true Ras binding domains II: in silico prediction based on homology modelling and energy calculations. Journal of molecular biology. 2005;348:759-75.
[3] Kiel C, Foglierini M, Kuemmerer N, Beltrao P, Serrano L. A genome-wide Ras-effector interaction network. Journal of molecular biology. 2007;370:1020-32.
[4] Wohlgemuth S, Kiel C, Krämer A, Serrano L, Wittinghofer F, Herrmann C. Recognizing and defining true Ras binding domains I: biochemical analysis. Journal of molecular biology. 2005;348:741-58.
[5] Amendola CR, Mahaffey JP, Parker SJ, Ahearn IM, Chen W-C, Zhou M, et al. KRAS4A directly regulates hexokinase 1. Nature. 2019;576:482-6.
[6] Schroder WA, Buck M, Cloonan N, Hancock JF, Suhrbier A, Sculley T, et al. Human Sin1 contains Ras-binding and pleckstrin homology domains and suppresses Ras signalling. Cellular signalling. 2007;19:1279-89.
[7] Chowdhury D, Hell JW. How CBP/Shank3 Guards Rap and H-Ras. Structure. 2020;28:274-6.
[8] Cai Q, Hosokawa T, Zeng M, Hayashi Y, Zhang M. Shank3 Binds to and Stabilizes the Active Form of Rap1 and HRas GTPases via Its NTD-ANK Tandem with Distinct Mechanisms. Structure. 2019.
[9] Ghai R, Bugarcic A, Liu H, Norwood SJ, Skeldal S, Coulson EJ, et al. Structural basis for endosomal trafficking of diverse transmembrane cargos by PX-FERM proteins. Proceedings of the National Academy of Sciences. 2013;110:E643-E52.
[10] The crystal structure of the RA domain of FLJ10324 (RADIL): http://www.rcsb.org/structure/3EC8.

Table S4. Human proteins containing RAS-related GTP-binding domain

No.	Entry	Protein name
1	P01112-1	HRAS1, p21HRAS
2	P01112-2	HRAS2, p19HRAS
3	P01111	NRAS
4	P01116-1	KRAS4A
5	P01116-2	KRAS4B, RASK2
6	Q7Z444	ERAS, KRAS2, HRASP
7	P11233	RALA
8	P11234	RALB
9	P10301	RRAS, RRAS1
10	P62070	RRAS2 TC21
11	O14807	RRAS3, MRAS
12	Q92963	RIT1, RIT, RIBB, ROC1
13	Q99578	RIT2, RIN, ROC2
14	P62834	RAP1A, KREV1
15	P61224	RAP1B
16	P61225	RAP2B
17	P10114	RAP2A
18	Q9Y3L5	RAP2C
19	Q15382	RHEB1
20	Q8TAI7	RHEB2
21	Q9Y272	RASD1, AGS1, DEXRAS1
22	Q96D21	RASD2, RHES, TEM2
23	O95057	DIRAS1, RIG, GBTS1
24	Q96HU8	DIRAS2
25	O95661	DIRAS3, ARHI, NOEY2, RHOI

Table S5. Dissociation constants determined for the RAS-effector interactions.

	HRAS	RRAS1	RAP1B	RAP2A	RALA	RHEB	RIT1
RASSF1	52±10	33±4	26±2	22±2	18±3	37±3	136±26
RASSF2	147±26	122±14	67±9	47±5	167±20	44±7	n.b.
RASSF3	500±164	435±61	116±26	100±18	139±15	64±9	n.b.
RASSF4	193±31	n.b.	101±21	88±10	191±46	47±7	58±9
RASSF5	1.0±0.1	56±4	4.0±1	2.0±0.2	49±10	46±7	n.b.
RASSF6	91±13	112±20	65±18	53±19	n.b.	56±13	98±27
RASSF7	140±67	30±5	72±13	68±9	101±30	76±16	34±3
RASSF8	n.b.	114±13	66±15	67±21	115±9	102±21	76±7
RASSF9	179±61	n.b.	74±18	66±15	n.b.	143±38	27±4
RASSF10	n.b.	99±10	73±6	67±16	n.b.	150±44	55±11
CRAF	0.3±0.1	3.3±1	30±7	n.b.	n.b.	35±9	139±40

Dissociation constants (K_d values ± SE calculated by matrix inversion using GraFit program) were determined by evaluating the fluorescence polarization data (Figures S5 and S6) shown in Figure 3 as bar charts. No binding (n.b.) stands for K_d values higher than 500 µM.

Table S6. Published structures of the RAS and Effector protein complexes.

Structures	PDB code	Res. (Å)	Ref.[a]
**RB domains**			
RAP1A-GTP-CRAF RB	1C1Y	2.2	[1]
RAP1A(E30D/K31E)-GppNHp-CRAF RB	1GUA	2.0	[2]
RAP1A(E30D/K31E)-GDP-CRAF RB(A85K/N71R)	3KUC	1.92	[3]
HRAS-GDP-CRAF-RB(A85K)	3KUD	2.15	[3]
HRAS-GppNHp-CRAF-RB	4G0N	2.45	[4]
HRAS(Q61L)-GppNHp-CRAF-RB	4G3X	3.25	[4]
KRAS-GppNHp-ARAF-RB	2MSE	NMR	[5]
HRAS(G12V)-GppNHp-PI3Kγ-RB(V223K/V326A)	1HE8	3.0	[6]
HRAS-GppNHp-Byr2-RB	1K8R	3.0	[7]
**RA domains**			
HRAS(D30E/E31K)-GppNHp-RASSF5-RA (L285M/K302D)	3DDC	1.8	[8]
HRAS(G12V)-GTP-GRAB14-RA/PH (K272A/E273A)	4K81	2.4	[9]
HRAS-GppNHp-RALGDS	1LFD	2.1	[10]
HRAS(G12V)-GTP-PLCε(Y2176L) RA2	2C5L	1.9	[11]
HRAS-GppNHp-Afadin RA1	6AMB	2.5	[12]
RAP1B-GppNHp-Rasip1 RA	5KHO	2.78	[13]

[a] References are listed below.

References

[1] Nassar N, Horn G, Herrmann CA, Scherer A, McCormick F, Wittinghofer A. The 2.2 Å crystal structure of the Ras-binding domain of the serine/threonine kinase c-Raf1 in complex with RaplA and a GTP analogue. Nature. 1995;375:554.

[2] Nassar N, Horn G, Herrmann C, Block C, Janknecht R, Wittinghofer A. Ras/Rap effector specificity determined by charge reversal. Nat Struct Biol. 1996;3:723-9.

[3] Filchtinski D, Sharabi O, Rüppel A, Vetter IR, Herrmann C, Shifman JM. What Makes Ras an Efficient Molecular Switch: A Computational, Biophysical, and Structural Study of Ras-GDP Interactions with Mutants of Raf. Journal of Molecular Biology. 2010;399:422-35.

[4] Fetics Susan K, Guterres H, Kearney Bradley M, Buhrman G, Ma B, Nussinov R, et al. Allosteric Effects of the Oncogenic RasQ61L Mutant on Raf-RBD. Structure. 2015;23:505-16.

[5] Mazhab-Jafari MT, Marshall CB, Smith MJ, Gasmi-Seabrook GMC, Stathopulos PB, Inagaki F, et al. Oncogenic and RASopathy-associated K-RAS mutations relieve membrane-dependent occlusion of the effector-binding site. Proceedings of the National Academy of Sciences. 2015;112:6625-30.

[6] Pacold ME, Suire S, Perisic O, Lara-Gonzalez S, Davis CT, Walker EH, et al. Crystal structure and functional analysis of Ras binding to its effector phosphoinositide 3-kinase gamma. Cell. 2000;103:931-43.

[7] Scheffzek K, Grunewald P, Wohlgemuth S, Kabsch W, Tu H, Wigler M, et al. The Ras-Byr2RBD complex: structural basis for Ras effector recognition in yeast. Structure. 2001;9:1043-50.

[8] Stieglitz B, Bee C, Schwarz D, Yildiz Ö, Moshnikova A, Khokhlatchev A, et al. Novel type of Ras effector interaction established between tumour suppressor NORE1A and Ras switch II. The EMBO journal. 2008;27:1995-2005.

[9] Qamra R, Hubbard SR. Structural Basis for the Interaction of the Adaptor Protein Grb14 with Activated Ras. PLoS One. 2013;8:e72473.

[10] Huang L, Hofer F, Martin GS, Kim SH. Structural basis for the interaction of Ras with RalGDS. Nat Struct Biol. 1998;5:422-6.

[11] Bunney TD, Harris R, Gandarillas NL, Josephs MB, Roe SM, Sorli SC, et al. Structural and Mechanistic Insights into Ras Association Domains of Phospholipase C Epsilon. Molecular Cell. 2006;21:495-507.

[12] Smith MJ, Ottoni E, Ishiyama N, Goudreault M, Haman A, Meyer C, et al. Evolution of AF6-RAS association and its implications in mixed-lineage leukemia. Nature communications. 2017;8:1099.

[13] Gingras AR, Puzon-McLaughlin W, Bobkov AA, Ginsberg MH. Structural basis of dimeric Rasip1 RA domain recognition of the Ras subfamily of GTP-binding proteins. Structure. 2016;24:2152-62.

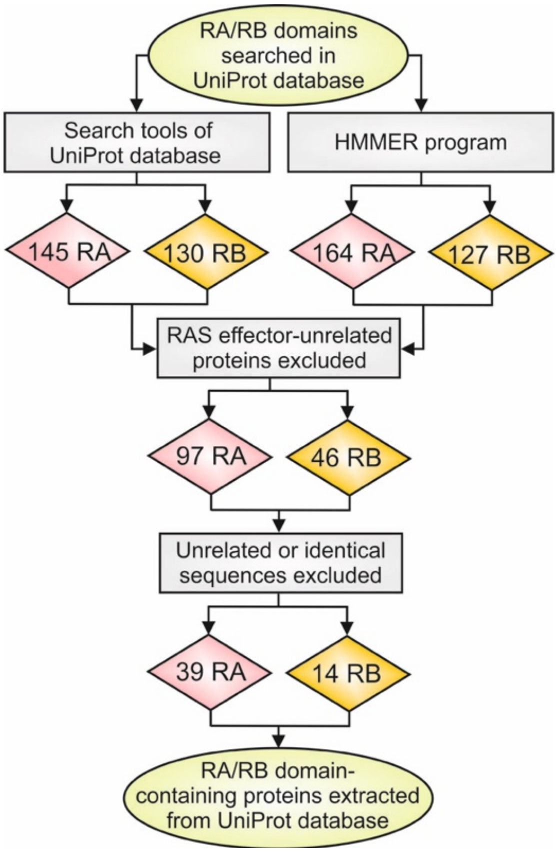

Figure S1. Flowchart of an *in-silico* search for RA/RB domains in data bases. RAS effector proteins in the human proteome were selected in a stepwise search. The initial search for input sequences, containing RA/RB domains, was performed in the UniProt database for proteins containing annotated RA/RB domains. Then HMMER was used to identify more sequence related domains. In the next step, the ClustalW sequence alignments of the output sequences were manually inspected and carefully processed to obtain all output sequences, such as 41 RA (in 39 different proteins) and 16 RB (in 14 different proteins) domains (Fig. S2 and S3), depicted in Tables S1 and S2.

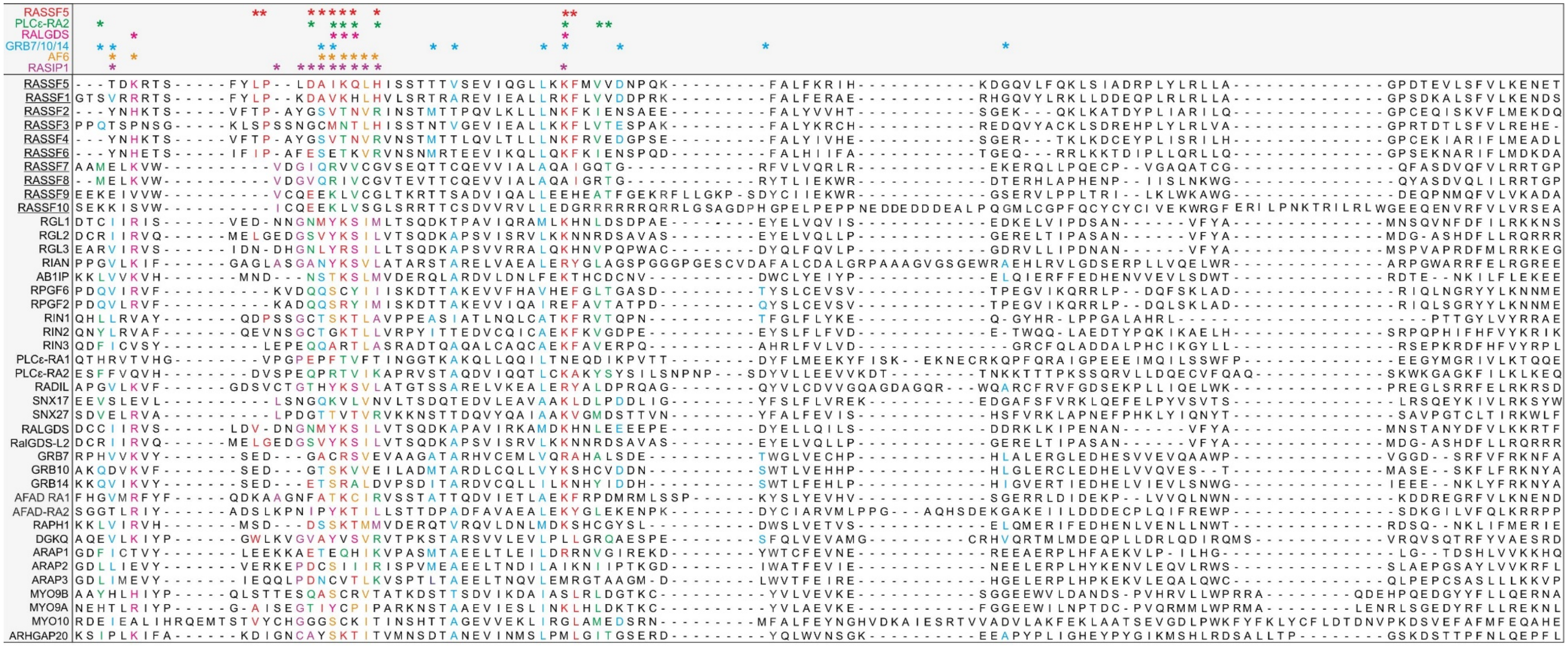

SIAS results: Seuqences identity of 9.2 % and similarity of 20.2 % for the RA domain of 39 proteins

Figure S2. Multiple sequence alignment of human RA domains. Amino acid sequences of 41 RA domains of 39 RA domain-containing proteins were aligned by using ClustalW and implemented in BioEdit with default multiple alignment parameters. Asterisks highlight RAS-binding amino acids of the respective effectors as indicated in red, green, magenta, blue, orange, and purple. Underlined proteins were biochemically investigated in this study.

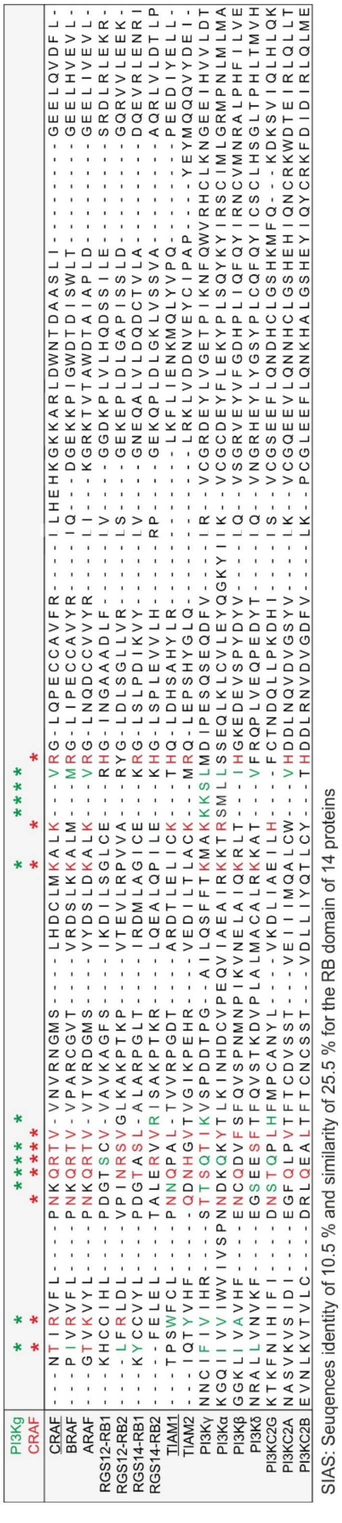

Figure S3. Multiple sequence alignment of human RB domains. Amino acid sequences of 16 RB domains of 14 RB domain-containing proteins were aligned by using ClustalW and implemented in BioEdit with default multiple alignment parameters. Asterisks highlight RAS-binding amino acids of the respective effectors as indicated in green and red. Underlined proteins were biochemically investigated in this study.

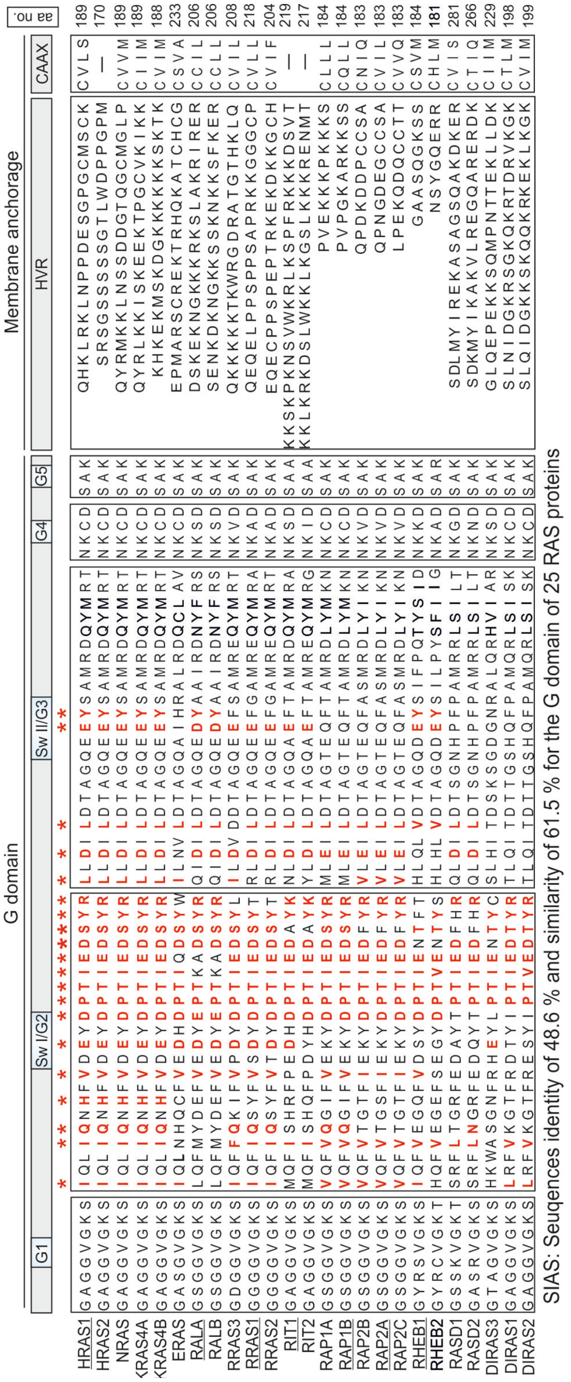

Figure S4. Multiple sequence alignment of human RAS protein family. Amino acid sequences of 25 RAS family proteins were aligned using ClustalW implemented in BioEdit with default multiple alignment parameters. Asterisks highlight effector-binding amino acids as indicated in red. Conserved signatures of the RAS proteins critical for GDP/GTP binding, GTP hydrolysis and proteins interactions are represented as G1 (or P loop for phosphate binding and magnesium ion coordination), G2 (or switch I for magnesium ion coordination and γ-phosphate binding), G3 (or switch II for γ-phosphate binding containing the catalytic glutamine), G4 (the major determinant of guanine base binding specificity) and G5 box (for guanine base binding). HVR (hypervariable region) and CAAX (C is cysteine, A is any aliphatic amino acid, and X is any amino acid) are critical motifs for association with the cell membrane. Underlined proteins were biochemically investigated in this study.

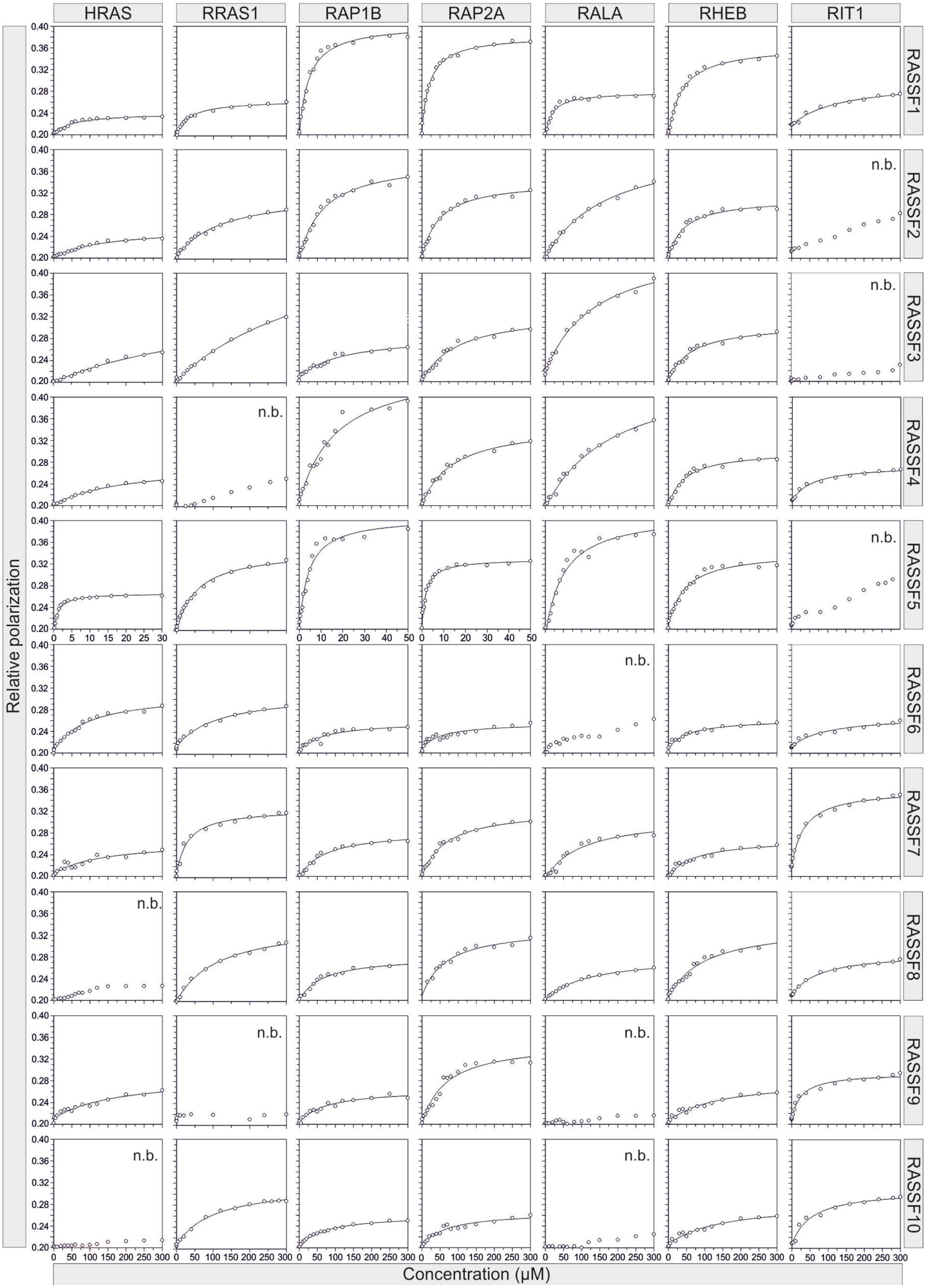

HRAS
RRAS1
RAP1B
RAP2A
RALA
RHEB
RIT1
RASSF1
RASSF2
RASSF3
RASSF4
RASSF5
RASSF6
RASSF7
RASSF8
RASSF9
RASSF10
n.b.
Relative polarization
Concentration (μM)

Figure S5. Fluorescence polarization measurements of RAS interactions with RASSF RA domains. Fluorescence polarization experiments were conducted to determine the dissociation constants (K_d) by titrating the active, mGppNHp-bound form of RAS proteins (1 μM) with increasing concentrations of the respective effector domains. The X-axis represents the concentration of the effector domain as MBP fusion proteins in μM and Y-axis represents fluorescence polarization. The lines through the data points indicate that equilibrium K_d values have been determined for the respective measurements. The K_d values are summarized in Figure 3 and Table S5.

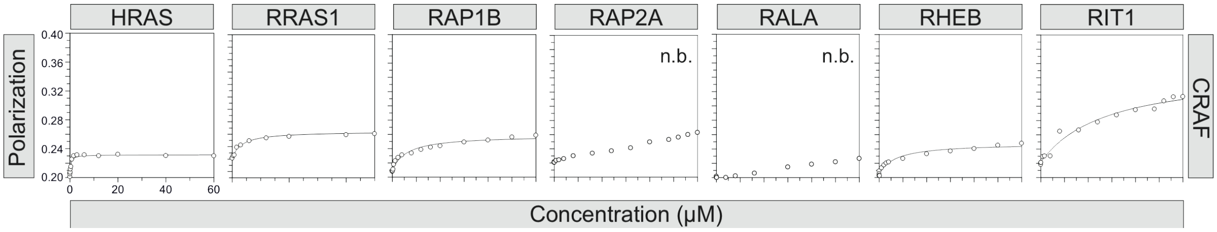

Figure S6. Fluorescence polarization measurements of RAS interactions with CRAF RB domain. Fluorescence polarization experiments were conducted to determine the dissociation constants (K_d) by titrating the active, mGppNHp-bound form of RAS proteins (1 µM) with increasing concentrations of CRAF RB domain. The X-axis represents the concentration of the effector domain as MBP fusion proteins in µM and Y-axis represents fluorescence polarization. The lines through the data points indicate that equilibrium K_d values have been determined for the respective measurements. The K_d values are summarized in Figure 3 and Table S5.

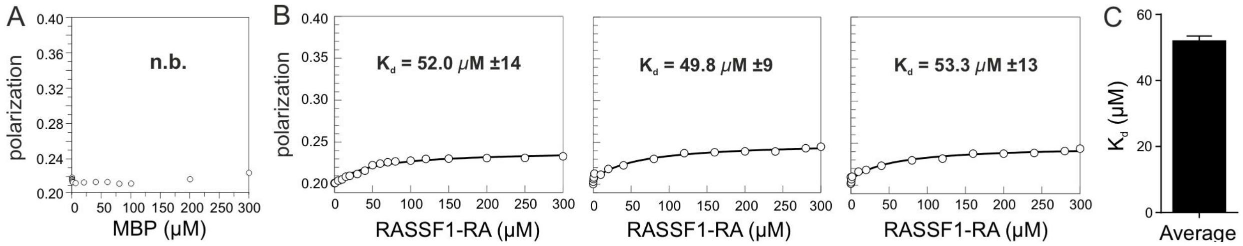

Figure S7. Control fluorescence polarization measurements. (A) Titration of mGppNHp-bound HRAS with purified MBP as a control experiment showed that MBP itself does not bind HRAS. (B) Three independent measurement for the interaction between HRAS•mGppNHp with RASSF1-RA resulted in very similar changes in fluorescence polarization. (C) The K_d values, determined for the three measurements in C, revealed a standard error of 1.021, which is far below the estimated standard error for the initial measurement in the manuscript (±10). SE was calculated using GraphPad Prism software. This Result allow the assumption that the standard error for each individual measurement in this study is the maximum probable error predicted for that measurement, which would be a lower number in triplicate measurements.

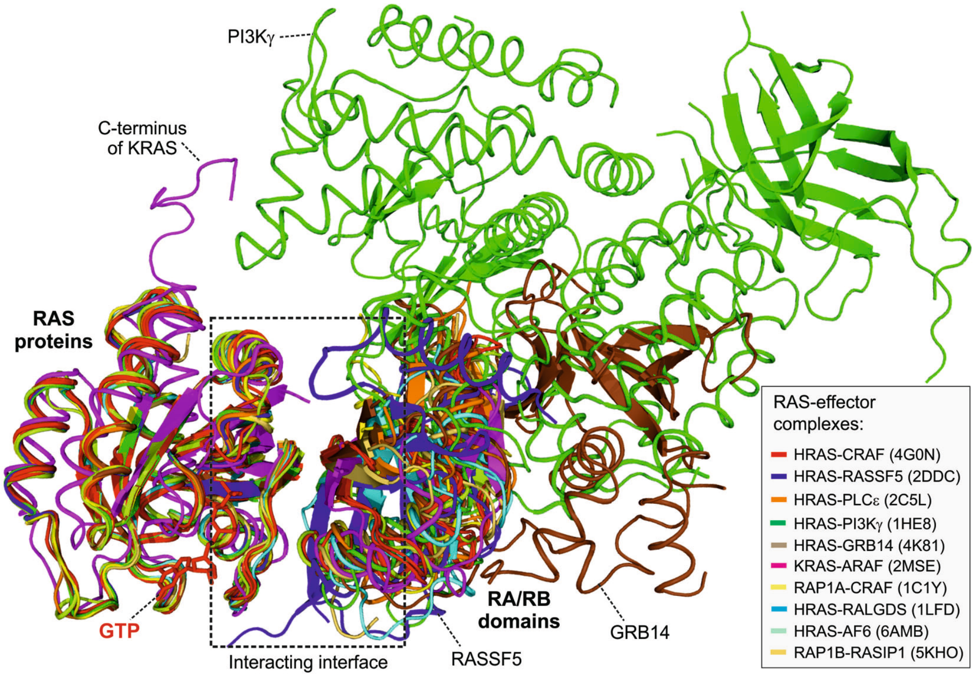

Figure S8. Superposition of all available RAS–effector complex structures. Ten structures of RAS-RA/RB domain complexes (see the inset and more details Table S6) were overlaid in ribbon representation. The interface residues (boxed), which were used for the generation of the matrix in Figure 3, are highlighted in Figure S9. Additional properties outside the interaction interface (box) are indicated.

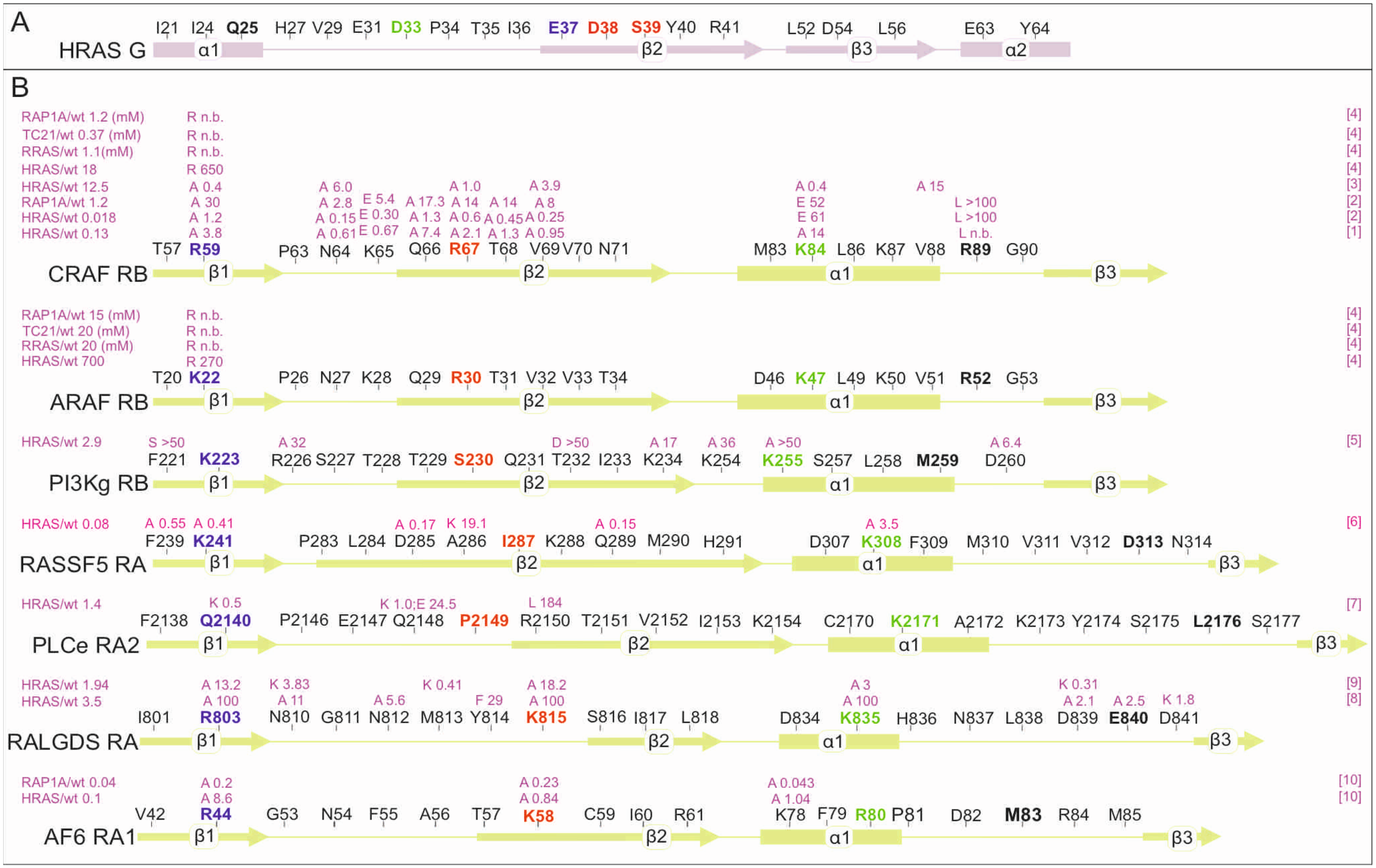
A
HRAS G
I21 I24 Q25 H27 V29 E31 D33 P34 T35 I36 E37 D38 S39 Y40 R41 L52 D54 L56 E63 Y64
α1 β2 β3 α2
B
RAP1A/wt 1.2 (mM) R n.b. [4]
TC21/wt 0.37 (mM) R n.b. [4]
RRAS/wt 1.1(mM) R n.b. [4]
HRAS/wt 18 R 650 [4]
HRAS/wt 12.5 A 0.4 A 6.0 A 1.0 A 3.9 A 0.4 A 15 [3]
RAP1A/wt 1.2 A 30 A 2.8 E 5.4 A 17.3 A 14 A 14 A 8 E 52 L >100 [2]
HRAS/wt 0.018 A 1.2 A 0.15 E 0.30 A 1.3 A 0.6 A 0.45 A 0.25 E 61 L >100 [2]
HRAS/wt 0.13 A 3.8 A 0.61 E 0.67 A 7.4 A 2.1 A 1.3 A 0.95 A 14 L n.b. [1]
T57 R59 P63 N64 K65 Q66 R67 T68 V69 V70 N71 M83 K84 L86 K87 V88 R89 G90
CRAF RB β1 β2 α1 β3
RAP1A/wt 15 (mM) R n.b. [4]
TC21/wt 20 (mM) R n.b. [4]
RRAS/wt 20 (mM) R n.b. [4]
HRAS/wt 700 R 270 [4]
T20 K22 P26 N27 K28 Q29 R30 T31 V32 V33 T34 D46 K47 L49 K50 V51 R52 G53
ARAF RB β1 β2 α1 β3
HRAS/wt 2.9 S >50 A 32 D >50 A 17 A 36 A >50 A 6.4 [5]
F221 K223 R226 S227 T228 T229 S230 Q231 T232 I233 K234 K254 K255 S257 L258 M259 D260
PI3Kg RB β1 β2 α1 β3
HRAS/wt 0.08 A 0.55 A 0.41 A 0.17 K 19.1 A 0.15 A 3.5 [6]
F239 K241 P283 L284 D285 A286 I287 K288 Q289 M290 H291 D307 K308 F309 M310 V311 V312 D313 N314
RASSF5 RA β1 β2 α1 β3
HRAS/wt 1.4 K 0.5 K 1.0;E 24.5 L 184 [7]
F2138 Q2140 P2146 E2147 Q2148 P2149 R2150 T2151 V2152 I2153 K2154 C2170 K2171 A2172 K2173 Y2174 S2175 L2176 S2177
PLCe RA2 β1 β2 α1 β3
HRAS/wt 1.94 A 13.2 K 3.83 K 0.41 A 18.2 A 3 K 0.31 [9]
HRAS/wt 3.5 A 100 A 11 A 5.6 F 29 A 100 A 100 A 2.1 A 2.5 K 1.8 [8]
I801 R803 N810 G811 N812 M813 Y814 K815 S816 I817 L818 D834 K835 H836 N837 L838 D839 E840 D841
RALGDS RA β1 β2 α1 β3
RAP1A/wt 0.04 A 0.2 A 0.23 A 0.043 [10]
HRAS/wt 0.1 A 8.6 A 0.84 A 1.04 [10]
V42 R44 G53 N54 F55 A56 T57 K58 C59 I60 R61 K78 F79 R80 P81 D82 M83 R84 M85
AF6 RA1 β1 β2 α1 β3

Figure S9. Secondary structures, the corresponding interacting residues, and published dissociation constants (K_d) for respective interactions are illustrated for HRAS G domain (A) and various RA/RB domains (B). The secondary structures and the corresponding interacting residues (above the secondary structures) were extracted from the structures deposited in the PDB (Table 6). Color codes of the interacting residues (blue, red, green, black; bold) correspond to those in Figures 3 and S2-S4. Determined K_d values for the Interaction of defined RA/RB domains with different RAS family proteins and the variants of the interacting residues are represented above the interacting residues, respectively. The numbers on the right side refer to the original studies (see references below).

References

[1] Block C, Janknecht R, Herrmann C, Nassar N, Wittinghofer A. Quantitative structure-activity analysis correlating Ras/Raf interaction in vitro to Raf activation in vivo. Nature structural biology. 1996;3:244-51.

[2] Nassar N, Horn G, Herrmann C, Block C, Janknecht R, Wittinghofer A. Ras/Rap effector specificity determined by charge reversal. Nature structural biology. 1996;3:723.

[3] Rudolph MG, Linnemann T, Grünewald P, Wittinghofer A, Vetter IR, Herrmann C. Thermodynamics of Ras/effector and Cdc42/effector interactions probed by isothermal titration calorimetry. Journal of Biological Chemistry. 2001;276:23914-21.

[4] Weber CK, Slupsky JR, Herrmann C, Schuler M, Rapp UR, Block C. Mitogenic signaling of Ras is regulated by differential interaction with Raf isozymes. Oncogene. 2000;19:169-76.

[5] Pacold ME, Suire S, Perisic O, Lara-Gonzalez S, Davis CT, Walker EH, et al. Crystal structure and functional analysis of Ras binding to its effector phosphoinositide 3-kinase γ. Cell. 2000;103:931-44.

[6] Stieglitz B, Bee C, Schwarz D, Yildiz Ö, Moshnikova A, Khokhlatchev A, et al. Novel type of Ras effector interaction established between tumour suppressor NORE1A and Ras switch II. The EMBO journal. 2008;27:1995-2005.

[7] Bunney TD, Harris R, Gandarillas NL, Josephs MB, Roe SM, Sorli SC, et al. Structural and mechanistic insights into ras association domains of phospholipase C epsilon. Molecular cell. 2006;21:495-507.

[8] Vetter IR, Linnemann T, Wohlgemuth S, Geyer M, Kalbitzer HR, Herrmann C, et al. Structural and biochemical analysis of Ras-effector signaling via RalGDS. FEBS letters. 1999;451:175-80.

[9] Kiel C, Selzer T, Shaul Y, Schreiber G, Herrmann C. Electrostatically optimized Ras-binding Ral guanine dissociation stimulator mutants increase the rate of association by stabilizing the encounter complex. Proceedings of the National Academy of Sciences. 2004;101:9223-8.

[10] Kiel C, Wohlgemuth S, Rousseau F, Schymkowitz J, Ferkinghoff-Borg J, Wittinghofer F, et al. Recognizing and defining true Ras binding domains II: in silico prediction based on homology modelling and energy calculations. Journal of molecular biology. 2005;348:759-75.

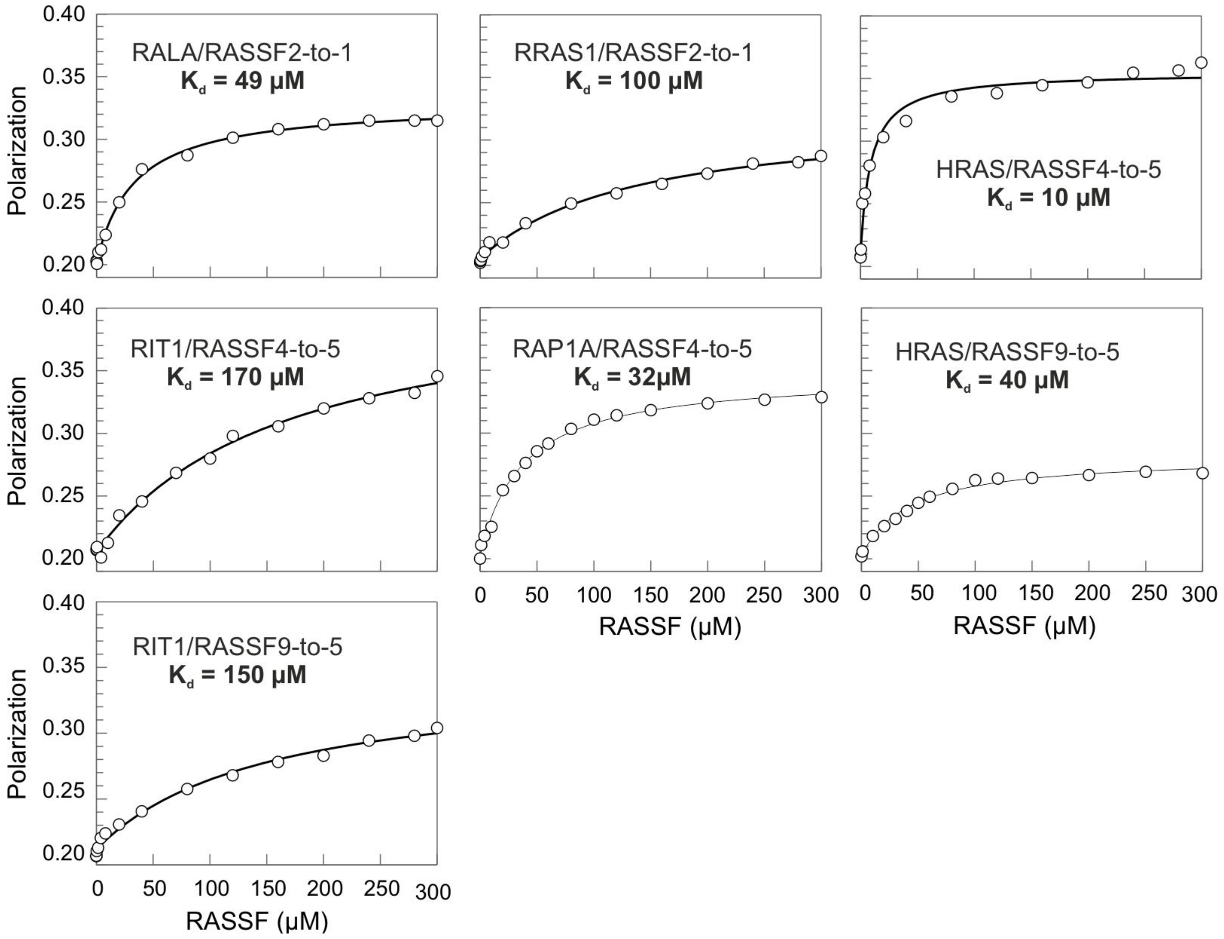

Figure S10. Fluorescence polarization measurements of the interaction of RASSF hotspot variants with various RAS family proteins. Fluorescence polarization experiments were conducted to determine the dissociation constants (K_d) by titrating the active, mGppNHp-bound form of RAS proteins (1 µM) with increasing concentrations of the RA domains of RASSF hotspot variants (RASSF2-to-1 A186K/Y187D/V190K/T191H, RASSF4-to-5: Y185D/S187I/V188K/ N188L and RASSF9-to-5: V40D/G42I/L43K/K45L/R46H; see Figure 3, boxed residues). The X-axis represents the concentration of the effector domain as MBP fusion proteins in µM and Y-axis represents fluorescence polarization. The lines through the data points indicate that equilibrium K_d values have been determined for the respective measurements. The K_d values are also summarized in Figure 4.

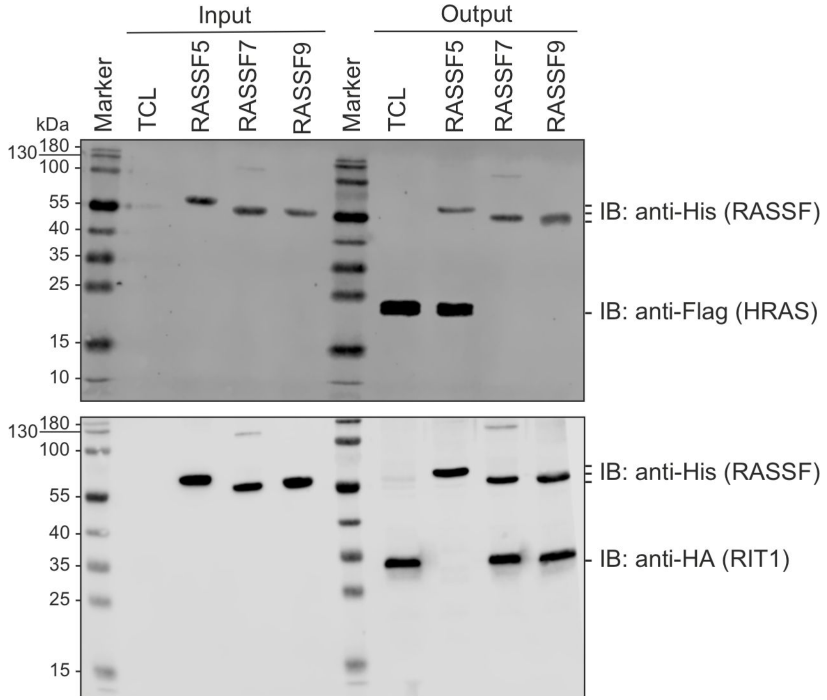

Figure S11. His-tag pull-down assay of RIT1 and HRAS binding by RASSF1-RA, RASSF7-RA and RASSF9-RA. HA-RIT1 and FLAG-HRAS, overexpressed in HEK 293T cells, were pulled down using His-tagged MBP-RA domains of RASSF5 (63 kDa), RASSF7 (54 kDa) and RASSF9 (55 kDa). Therefore, His-tagged RASSF-RA proteins (20 µg per protein and experiment) were first coupled to the Ni-NTA beads (100 µl per experiment) before mixing them with the cell lysates (200 µg per experiment). Note that His-pulldown assay did not work when cell lysate and the His-tagged proteins were added to the bead at the same time. Immunoblots of total cell lysates (TCL), evaluated using an Odyssey Fc Imaging System (LI-CORE Biosciences), were served as loading control to detect HA-RIT1 and FLAG-HRAS, respectively, analyzed by immunoblotting (IB) using anti-HA (SC-805, Santa Cruz) and anti-FLAG (F7425, Sigma) antibodies. Input samples represent the quantity of the RASSF proteins prior to being added to the beads and output samples indicate the quantity of the bound RASSF proteins to the beads after pull down. An anti-His (RM146, Thermo Fisher) antibody was used for detection of His-tagged RASSF5, RASSF7 and RASSF9 in input and output as loading control. Input and output samples showed that the RASSF proteins are comparably bound to the beads.
